# Anisotropy vs isotropy in living cell indentation with AFM

**DOI:** 10.1038/s41598-019-42077-1

**Published:** 2019-04-08

**Authors:** Yuri M. Efremov, Mirian Velay-Lizancos, Cory J. Weaver, Ahmad I. Athamneh, Pablo D. Zavattieri, Daniel M. Suter, Arvind Raman

**Affiliations:** 1 School of Mechanical Engineering, Purdue University, West Lafayette, Indiana USA; 2 Birck Nanotechnology Center, Purdue University, West Lafayette, Indiana USA; 3 Lyles School of Civil Engineering, Purdue University, West Lafayette, Indiana USA; 4 Department of Biological Sciences, Purdue University, West Lafayette, Indiana USA; 5 Bindley Bioscience Center, Purdue University, West Lafayette, Indiana USA; 6 Purdue Institute for Integrative Neuroscience, West Lafayette, Indiana USA; 7 Present Address: University of South Carolina, Department of Biological Sciences, Jones PSC Building, 712 Main Street, room 517, Columbia, SC 29208 USA

**Keywords:** Characterization and analytical techniques, Applications of AFM, Biophysics, Biomaterials - cells

## Abstract

The measurement of local mechanical properties of living cells by nano/micro indentation relies on the foundational assumption of locally isotropic cellular deformation. As a consequence of assumed isotropy, the cell membrane and underlying cytoskeleton are expected to locally deform axisymmetrically when indented by a spherical tip. Here, we directly observe the local geometry of deformation of membrane and cytoskeleton of different living adherent cells during nanoindentation with the integrated Atomic Force (AFM) and spinning disk confocal (SDC) microscope. We show that the presence of the perinuclear actin cap (apical stress fibers), such as those encountered in cells subject to physiological forces, causes a strongly non-axisymmetric membrane deformation during indentation reflecting local mechanical anisotropy. In contrast, axisymmetric membrane deformation reflecting mechanical isotropy was found in cells without actin cap: cancerous cells MDA-MB-231, which naturally lack the actin cap, and NIH 3T3 cells in which the actin cap is disrupted by latrunculin A. Careful studies were undertaken to quantify the effect of the live cell fluorescent stains on the measured mechanical properties. Using finite element computations and the numerical analysis, we explored the capability of one of the simplest anisotropic models – transverse isotropy model with three local mechanical parameters (longitudinal and transverse modulus and planar shear modulus) – to capture the observed non-axisymmetric deformation. These results help identifying which cell types are likely to exhibit non-isotropic properties, how to measure and quantify cellular deformation during AFM indentation using live cell stains and SDC, and suggest modelling guidelines to recover quantitative estimates of the mechanical properties of living cells.

## Introduction

Recent developments in fluorescent live-cell imaging and biophysical methods have significantly advanced our understanding of the dynamic biochemical and mechanical processes underlying cellular functions such as cell migration. These cellular functions are intimately related to mechanical properties of live cells such as stiffness and adhesion. Thus, linking cell mechanical properties to specific cellular structures is of high interest to many cell biologists. Atomic Force Microscope (AFM)-based indentation of live cells is one of the most frequently used techniques to assess mechanical properties of cells due to its relative ease of operation, high precision of force measurement, and high spatial resolution^1–4^. Mathematical models of contact mechanics between the AFM tip and the cell^5–11^ are required to interpret and quantify data derived from AFM indentation on live cells. Isotropic mechanical response is a common underlying assumption in these models.

However, without the visualization of the cell structure and geometry of deformation simultaneously during cell indentation, it is extremely difficult, if not impossible, to verify if many underlying assumptions of the model are actually met. Such simultaneous visualization can help assess how the inhomogeneity of the cell structure affects the indentation; how the underlying cytoskeleton behaves to produce observed cellular mechanical behaviour; and to check the presence of any effects of the indentation on cells, like distant cytoskeletal rearrangements, residual damage or induced mechanoresponse^12–24^.

Here, we integrated the AFM with a spinning disk confocal (SDC) microscope to create an experimental platform for simultaneous analysis of cellular deformation and mechanical properties with high spatio-temporal resolution^15–17,25^. With live-cell imaging stains to fluorescently label the F-actin and microtubule cytoskeleton as well as the plasma membrane, we were able to directly observe structural changes during the indentation process with a spherical indenter in NIH 3T3 fibroblasts and MDA-MB-231 epithelial cancer cells. We found a strong correlation between presence of the perinuclear actin cap fibers and cell mechanical properties; highly anisotropic indentation geometry was found in cells with actin cap. To further assess anisotropy in cell mechanical properties, we performed finite element simulations and compared with the experimental surface displacement data. Our observations suggest a significant role of an anisotropic deformability and stiffness in the mechanics of cells.

## Results

### Cell viscoelastic properties and the effect of live-cell imaging stains

**Figure 1:**
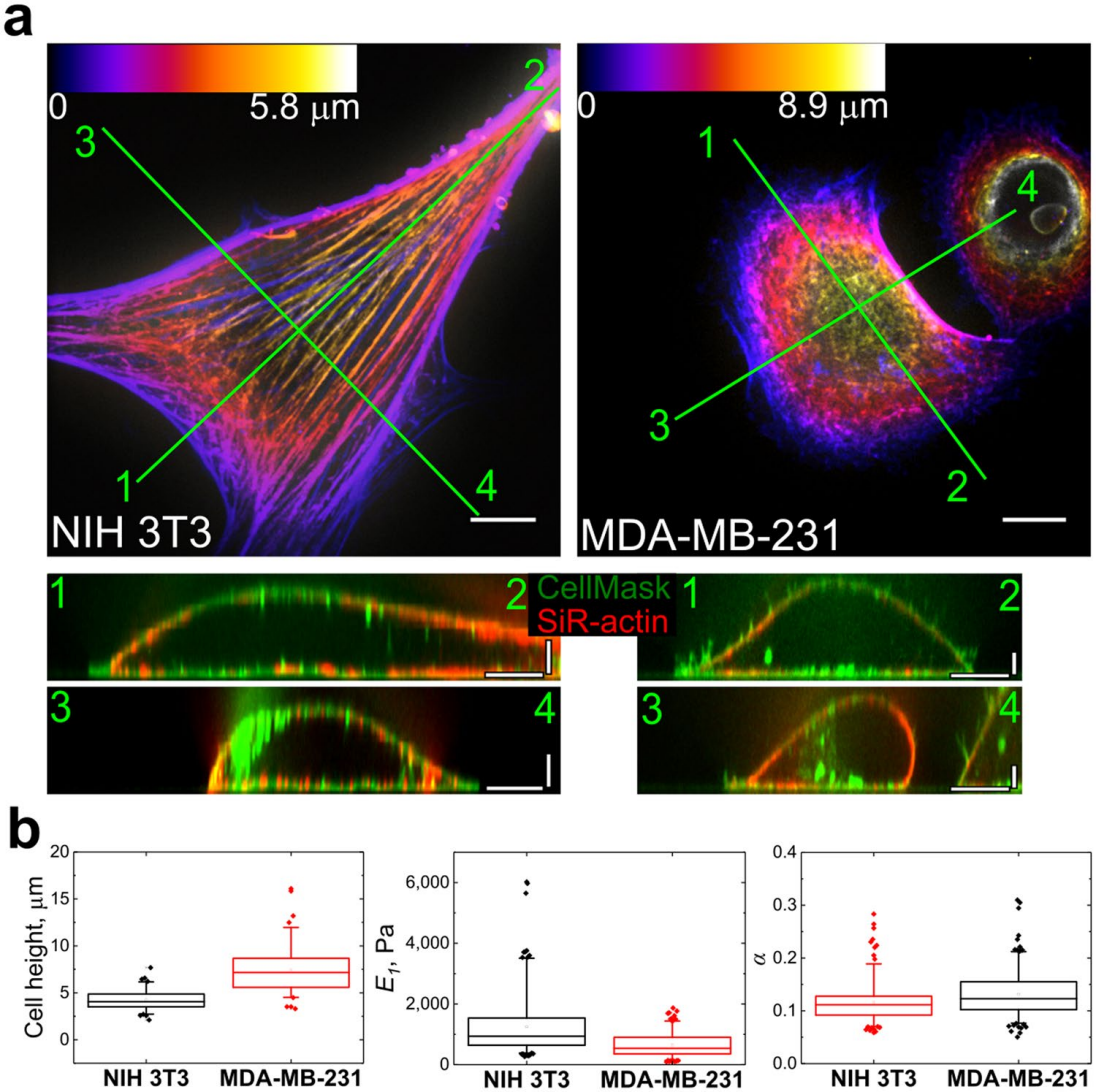
Morphology and mechanical properties of fibroblasts and cancer cells. (**a**) F-actin (SiR-actin) structure in NIH 3T3 and MDA-MB-231 cells, the height is colour coded with respect to the scaling shown in the colour scale bars. Vertical cross-sections along the marked lines shows that F-actin is mostly localized in the submembranous region (CellMask staining for plasma membrane). Scale bars 10 μm in the horizontal direction and 2 μm in the vertical direction. (**b**) Box plots of cell height, Young’s relaxation modulus scale factor *E*_1_, and power-law exponent *α*. The differences between all distributions are significant at the p < 0.001 level.

Live cell imaging requires special fluorescent dyes, some of which were shown to alter properties of their targeted structures and overall cell mechanical properties^26–28^. Among all stains used, only SiR-actin caused significant cell stiffening (the details are given in Supplementary Information, Section C, Table S1 and Fig. S1). For viscoelastic characterization, the power law rheology model (Eq. 3) was selected because it has been shown to sufficiently describe cell properties in a wide range of indentation times^29,30^. *E*_1_ is the relaxation modulus at *t* = 1 s (scale factor of the relaxation modulus), characterizing the stiffness of the sample; *α* is the power law exponent determining the relaxation behaviour. As expected, NIH 3T3 fibroblasts were more spread, flatter (mean height of 4.2 ± 1.1 μm, n = 83 vs 7.4 ± 2.5 μm, n = 80, p < 0.001), stiffer (*E*_1_ = 1.3 ± 0.9 kPa, n = 90 vs 0.6 ± 0.4 kPa, n = 67, p < 0.001), and more solid-like (*α* = 0.12 ± 0.4, n = 90 vs *α* = 0.14 ± 0.4, n = 67, p < 0.001) when compared to cancer MDA-MB-231 cells (Fig. 1b, Table S1). The question arises which cell component is most responsible for this difference in mechanical properties. Observing significant differences in F-actin organization between the two cell types and based on the majority of previous studies^4,31–34^, here we concentrated our attention on the organization of the F-actin cytoskeleton in these cells. In NIH 3T3 fibroblasts, most of the F-actin was found in the form of stress fibers (on both apical and basal sides of the cell), whereas MDA-MB-231 cells exhibited a more irregular and disorganized F-actin network located underneath the plasma membrane (actin cortex) (Fig. 1a), which is in agreement with previous reports for different types of cancer cells^35,36^.

### Presence of perinuclear actin cap fibers correlate with the increased cell stiffness

**Figure 2:**
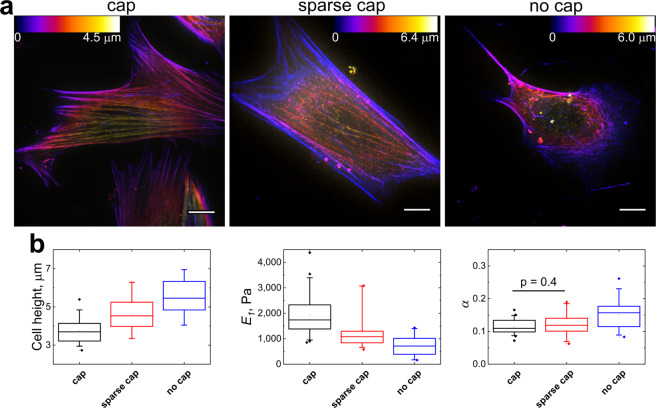
Correlation between perinuclear actin cap structure and mechanical properties of NIH 3T3 fibroblasts. (**a**) Typical distribution of actin in fibroblasts with well-developed actin cap (cap), sparse cap, and with no cap (SiR-actin staining). The height is colour coded with respect to the scaling shown in the colour scale bars. Scale bars 10 μm. (**b**) Box plots of cell height, Young’s relaxation modulus scale factor *E*_1_, and power-law exponent *α* (Data for the cells with SiR-actin staining). The differences between all distributions except the one marked in the last panel are significant at the p < 0.01 level.

In agreement with previous studies, perinuclear actin cap fibers (previously also referred as apical stress fibers^32,33,37^) could be distinguished in most of the NIH 3T3 fibroblasts (≈80%)^38^. These stress fibers aligned with the long axis of fibroblasts emanate from one edge of the cell at the basal side, extend over the nucleus, and terminate in adhesions at the opposite cell edge forming dome-like structure^39,40^. Actin cap fibers link to the nucleus via LINC (Linker of Nucleoskeleton and Cytoskeleton) complexes and regulate the shape of the nucleus during interphase; actin cap fibers also participate in fast mechanotransduction by transducing force from the cell environment to the nucleus^40–42^. Following previous work^32,38,43^, we classified NIH 3T3 cells into 3 types based on the level of development of the perinuclear actin cap. The first group had a well-developed actin cap with dense thick fibers (“cap” group, ≈50 ± 5% of the total population), the second group had a less-developed cap with sparse thinner fibers (“sparse cap”, ≈30 ± 5%), and the third group consisted of cells lacking actin cap fibers (“no cap”, ≈20 ± 5%) (Fig. 2a). The percentage of cells in different groups was confirmed with both live cell actin stains and fixed cells stained with phalloidin (Fig. S2). The combined AFM/SDC data (with SiR-actin or Cell-Light^TM^ Actin-GFP BACMAM 2.0 staining) showed that cells with developed actin cap are about 3 times stiffer than the cells without cap and have a lower height (Fig. 2b, Tables S2 and S3) in agreement with previous studies^32,33^. Moreover, the viscoelastic analysis showed that cells with the actin cap also had a lower power law exponent, which makes them more solid-like. On the other hand, the density of the ventral stress fibers did not noticeably correlate with the density of the actin cap and the cell stiffness. Therefore, the large heterogeneity in measured cell mechanical properties for the population of fibroblasts could at least in part be explained by the heterogeneity in the structure of the F-actin cytoskeleton, specifically the level of cap development. MDA-MB-231 cells did not have a pronounced actin cap and, therefore, most of the cells could be referred to the “no cap” group. Interestingly, mechanical properties of MDA-MB-231 cells were quite close to the properties of NIH 3T3 cells from the “no cap” group (Tables S1 and S2). In the following section, we studied how the presence of actin cap fibers can affect the indentation and lead to the higher cell stiffness.

### Anisotropic indentation pattern emerges due to presence of the actin cap

**Figure 3:**
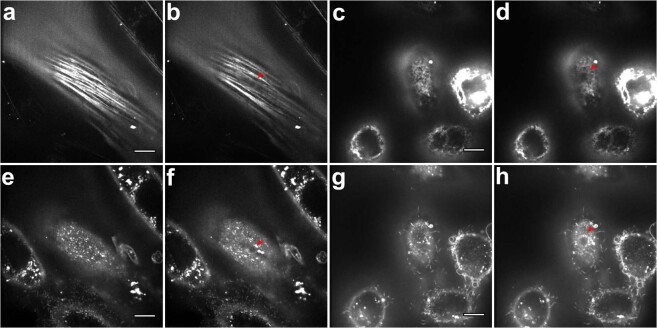
Deformation of single apical stress fibers in NIH 3T3 fibroblasts (**a**,**b**) causes anisotropic (non-axisymmetric) indentation profile (**e**,**f**), while isotropic indentation profile is presented in MDA-MB-231 cells (**c,d**,**g**,**h**). Single-plane recording experiment (protocol 2, see Supplementary Information, Section B). SiR-Actin (**a**–**d**) and CellMask (**e**–**h**) staining for F-actin and the plasma membrane, respectively. The cantilever is above the cell in (**a**,**e**,**c**,**g**); the bead indents the cell in (**b**,**d**,**f**,**h**) and its location is marked with red triangles. The perinuclear actin cap fibers located underneath the bead deformed most, going deeper out of the focal plane (**b**). Anisotropic deformation pattern was also observed with membrane staining as a decrease in the dye intensity along the fiber direction and an extension in the perpendicular direction (**f**). In MDA-MB-231 cells, isotropic deformation pattern was observed with both stainings revealing a circular indentation profile. Scale bars 10 μm.

Next, we directly observed AFM indentation with SDC microscope. In the fast single-plane recording experiments (protocol 2, see Supplementary Information, Section B, and Fig. S3) most notable observation was the deformation of single perinuclear actin cap fibers in NIH 3T3 fibroblasts (Fig. 3A,B, Movie S1). During the indentation, the cap fibers located underneath the spherical (5 µm diameter) probe deformed the most, leading to anisotropic indentation pattern, observed also with membrane staining (Fig. 3e,f). While the cap fibers pressed deeper into the cell due to the action of the bead, the cell deformed and bulged in the perpendicular direction to conserve the volume. Overall, this resulted in a contact periphery to be elliptical rather than circular (Fig. 3f). In cancerous MDA-MB-231 cells, which mostly lacked well-developed actin cap, as well as in NIH 3T3 cells from “no cap” group, the deformation was more isotropic (Fig. 3d,h; Movies S2 and S3) with close to circular contact periphery.

**Figure 4:**
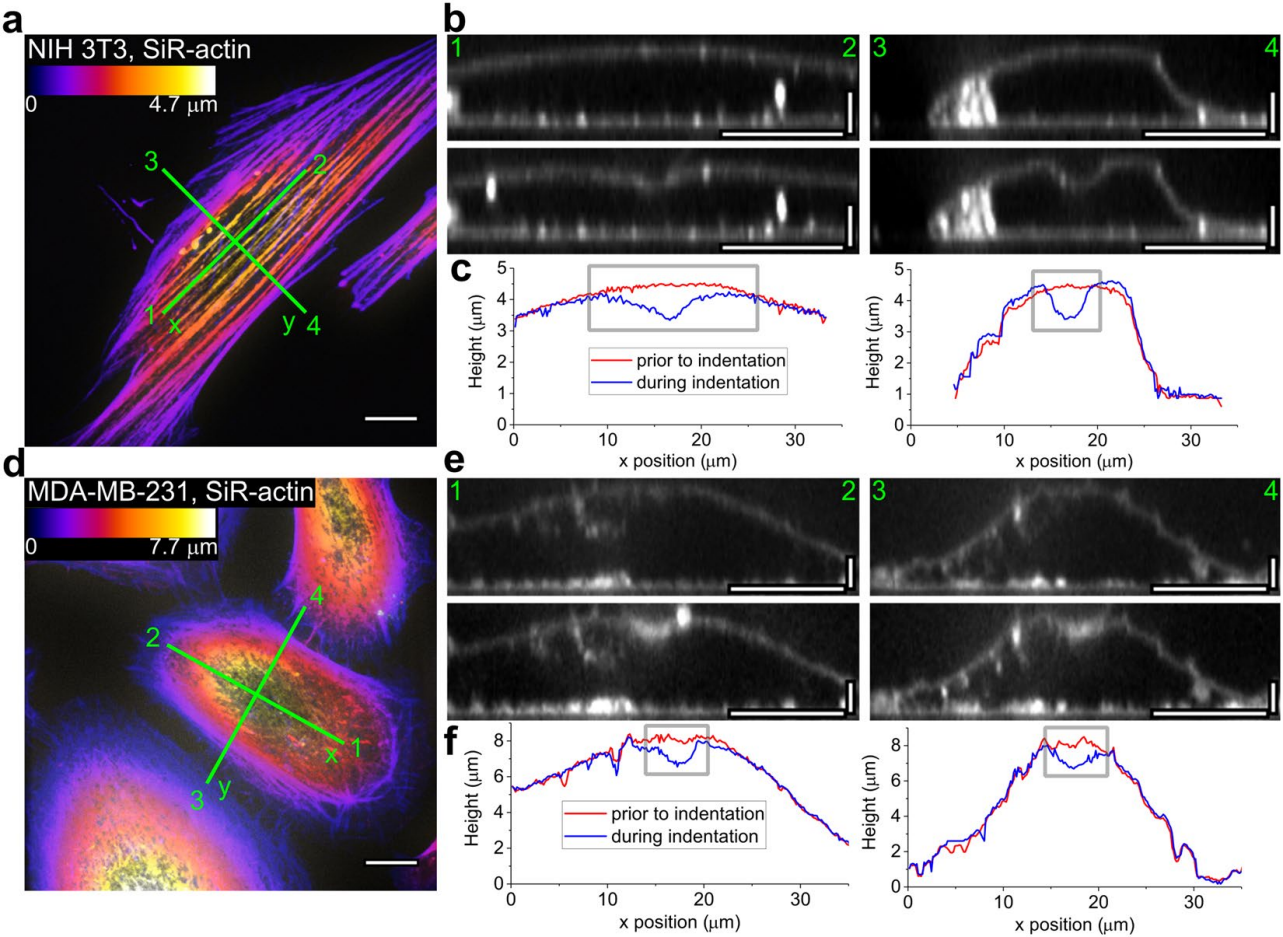
Anisotropic and isotropic indentation profiles in NIH 3T3 (**a**–**c**) and MDA-MB-231 (**d**–**f**) cells, respectively. Full z-stacks were obtained before and during the hold period (protocol 1, see Supplementary Information, Section B). SiR-actin staining (**a**,**d**) reveals actin stress fiber orientation and CellMask staining (**b**,**e**) shows the membrane deformation (surface displacement profile). (**a**,**d**) Colour coded z-projections of the SiR-actin staining. (**b**,**e**) Reconstructed vertical cross-sections along the marked lines, before (top) and during (middle) indentation. (**c**,**f**) The calculated position of the membrane showing the anisotropic indentation profile in NIH 3T3 cells but not in MDA-MB-231 cells. Scale bars: 10 μm for the z-projections; 10 μm in the horizontal direction and 2 μm in the vertical direction for the cross-sections.

To confirm the presence of the non-axisymmetric indentation profile, we obtained full z-stacks before indentation and during the hold period (protocol 1, see Supplementary Information, Section B), thus assessing 3D changes in cell morphology during the indentation process. The non-axisymmetric deformation pattern could be noticed on NIH 3T3 fibroblasts when comparing cross-sections along and perpendicular to the main cell axis (and, therefore, the direction of the actin cap stress fibers) (Fig. 4a–c). In the cross-sections along the apical cap fibers, the indentation profile was more elongated, while in the perpendicular cross-section it conformed more closely with the probe geometry. MDA-MB-231 cells had generally an axisymmetric indentation profile, which correlates with the absence of the actin cap (Fig. 4d–f). The same observations were obtained from a partial Z-stacks (protocol 3, see Supplementary Information, Section B, Fig. S4).

**Figure 5:**
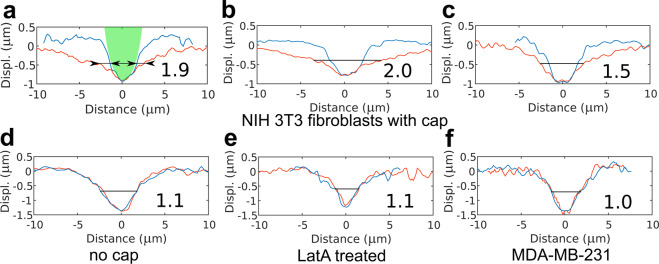
Examples of the surface displacement profiles for the different cells. The red line is the profile along the apical cap fibers and/or the main cell axis, the blue line is the profile perpendicular to it. The number near the profile is the calculated degree of anisotropy (D.A.). Surface displacement profiles for NIH 3T3 fibroblasts with a well-developed actin cap (**a–c**), without actin cap (**d**), after Latrunculin A (LatA) treatment (**e**) and typical for MDA-MB-231 cell (**f**) are presented. In (**a**), the microsphere used as an AFM probe is shown with green for the reference (it has an elliptical shape due to different *x-y* scaling), and two widths measured at half-depth for calculation of the D.A. are shown with arrows.

To assess the degree of anisotropy in the indentation, first, the surface displacement profile was calculated by subtracting the plasma membrane profile during the indentation from the one before the indentation. In this way, two surface displacement profiles were obtained, one along the direction of the apical cap fibers and another one perpendicular to it. If apical cap fibers were absent the direction of the main cell axis was selected to extract the surface displacement profile. Next, we introduced a dimensionless parameter, the degree of anisotropy (D.A.), that is the ratio of the larger width of the surface displacement profile to the smaller width measured at half-depth (Fig. 5a). Larger D.A. corresponds to a larger difference between profiles along perpendicular directions and therefore, more anisotropic cell properties. The D.A. parameters calculated for different cells are presented in Fig. 5 and Table 1. From these data it is clearly seen that degree of anisotropy correlates with the presence and level of development of the perinuclear actin cap. Low D.A. values were found in the fibroblasts without the cap fibers (Fig. 5d) and MDA-MB-231 cells (Fig. 5f). Application of actin fluorescent stains did not significantly change the average values of D.A., although a slight tendency for its increase was noticed, especially for SiR-actin probe (Table 1).

**Table 1:** Degree of anisotropy (D.A.) measured for different cell populations based on the surface displacement profiles.

		D.A.	
NIH-3T3	w/o actin stain	1.6 ± 0.4 (n = 18)	
	SiR-actin	1.8 ± 0.6 (n = 25)	cap: 2.1 ± 0.5 (n = 15) sparse cap: 1.4 ± 0.2 (n = 4) no cap: 1.2 ± 0.2 (n = 6)
	Actin-GFP	1.7 ± 0.5 (n = 25)	cap: 2.1 ± 0.5 (n = 13) sparse cap: 1.5 ± 0.2 (n = 9) no cap: 1.1 ± 0.1 (n = 3)
	After LatA treatment	1.1 ± 0.1 (n = 15)	
MDA-MB-231	w/o actin stain	1.2 ± 0.2 (n = 13)	
	SiR-actin	1.1 ± 0.1 (n = 12)	
	Actin-GFP	1.1 ± 0.1 (n = 15)	

In the case when cells were stained for actin, the D.A. for subgroups (cap, sparse cap, no cap) is also presented. Mean ± s.d.

To further confirm the dependence of the D.A. from the presence of the actin cap, we disrupted it with a low-dose treatment (100 nM) of the F-actin depolymerizing drug latrunculin A in NIH 3T3 fibroblasts. Indeed, after the treatment, we observed low values of the D.A. corresponding to the isotropic material (Fig. 5E), together with a major decrease in stiffness (~90% in *E*_1_), growth of power law exponent (~50%), and increase in cell height (~40%) (Fig. S5A,C). Notably, as reported in the previous studies, ventral stress fibers mostly preserved after the treatment (Fig. S5B), which emphasizes the importance of 3D actin cytoskeleton structure and particularly actin cap in cell mechanics.

### Assessment of anisotropy in cell mechanical properties by finite element simulations

**Figure 6:**
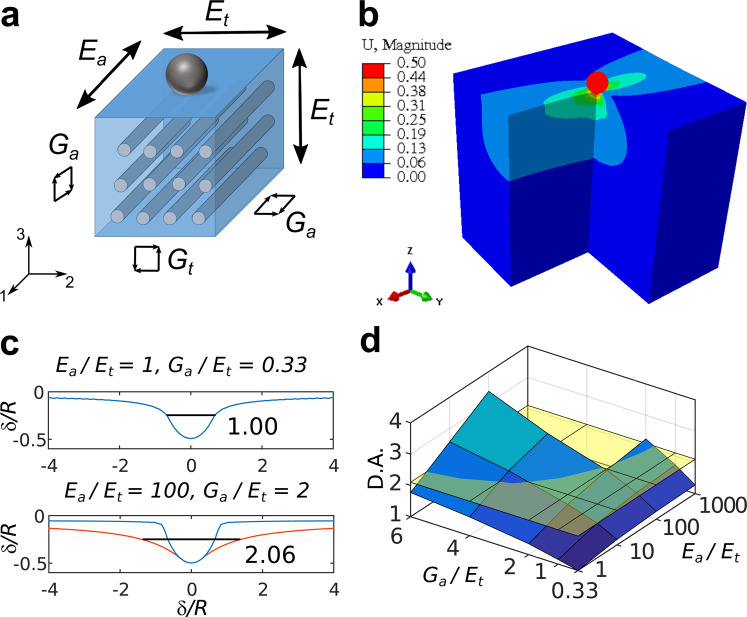
Finite element simulation of the indentation of the transversely isotropic material. (**a**) A schematic representation of the transversely isotropic material. Subscripts “a” and “t” are used for axial and transverse material properties respectively. Indentation axis is parallel to the plane of isotropy. (**b**) Vertical displacement obtained with the three-dimensional finite element model for incompressible transversely isotropic material with *E*_*a*_/*E*_*t*_ = 100 and *G*_*a*_/*E*_*t*_ = 4. A quarter of the geometry has been cut to show the displacement field inside the material. (**c**) Examples of the surface displacement profiles for material with different parameter ratios. The value of D.A. is shown near profiles. (**d**) The surface plot of the degree of anisotropy (D.A.) versus *G*_*a*_/*E*_*t*_ (linear scale) and *E*_*a*_*/E*_*t*_ (log scale) ratios. *G*_*a*_/*E*_*t*_ ratio affects D.A. stronger than *E*_*a*_*/E*_*t*_ ratio, while the combined effect is even more significant. The average D.A. value for the fibroblasts with actin cap fibers (2.1) is marked with the yellow plane.

Mechanical anisotropy of elasticity can be characterized using a large number of independent parameters. Here, we explored whether one of the simplest models for anisotropy with a small number of independent parameters can in fact explain the observed membrane deformation profiles on cells with actin cap fibers. For this, we performed finite element simulations on a 3D elastic transversely isotropic body, which can be considered as a material with a single family of homogenously distributed and aligned fibers^44,45^ (Fig. 6a). With the additional assumption of material incompressibility, this model describes mechanical anisotropy with only three independent parameters: Young’s moduli *E*_*t*_ and *E*_*a*_, where subscripts “a” and “t” are used for axial (along the fiber direction) and transverse material properties, and an in-plane shear modulus *G*_*a*_ (see Supplementary Information, Section D for details). The FE model was constructed according to the following considerations (Fig. 6b): fibers, corresponding to the actin cap stress fibers in the cell, are located parallel the substrate, as could be approximated in a vicinity of the indentation area. Indentation axis is parallel to the plane of isotropy and perpendicular to the fibers. Fibers are stiffer than the surrounding matrix (cytoplasm), therefore *E*_*a*_ and *G*_*a*_ are higher than *E*_*t*_ and *G*_*t*_ respectively, where *G*_*t*_ = *E*_*t*_/(4 − *E*_*t*_/*E*_*a*_). Thus, we varied *E*_*a*_/*E*_*t*_ from 1 (isotropic case) to 1000 and *G*_*a*_/*E*_*t*_ (which is proportional to *G*_*a*_/*G*_*t*_) from 0.33 (isotropic case) to 6 and analysed the corresponding surface displacement profiles same way as we did for cells to obtain the D.A. parameter (Fig. 6c). Besides this, the “effective isotropic” indentation modulus *E*_*eff*_ was calculated for the simulated force curves (see Supplementary Information, Sections E and G, Fig. S6). The D.A. was in range from 1 (isotropic case) to 3.37 (highly anisotropic case), thus covering the range observed experimentally on cells (Fig. 6d). Ancillary simulations performed on a cell-like shaped object (an ellipsoidal cap) demonstrated that the D.A. is only weakly affected by the geometry (see Supplementary Information, Section E for details).

The simulation showed, that it is impossible to determine exact values of all three parameters *E*_*a*_, *E*_*t*_, and *G*_*a*_ based only on simulated force curves and surface displacement profiles: very close curves and profiles could be caused by different sets of the mechanical parameters (Fig. S7D,E). The simulations showed that the high D.A. values may originate from either high *G*_*a*_/*E*_*t*_, very high *E*_*a*_/*E*_*t*_ ratio, or some combination of both (Figs 6d and S7).

To determine which of the *G*_*a*_/*E*_*t*_, very high *E*_*a*_/*E*_*t*_ ratios is more important for high D.A. values and to obtain lower and higher estimates for both of them, we combined FE data with the numerical analysis based on the rule of mixtures^46,47^ for fiber-reinforced composite materials (see Supplementary Information, Section F and Fig. S8 for details). This analysis indicated that highly anisotropic cells with the observed value of D.A. = 2.1 could have high *E*_*a*_/*E*_*t*_ ratios (from ≈400 to ≈100000) and relatively small *G*_*a*_/*E*_*t*_ ratios (from 0.33 to ≈1.5). The estimation of exact *G*_*a*_ and *E*_*a*_ moduli, however, requires additional information about the properties of the perinuclear stress fibers and/or some independent mechanical measurements. These could be, for example, in-plane measurements of the forces and displacements with magnetic tweezers^48^ or use of asymmetric indenter with indentation before and after 90 degrees indenter rotation^49^. However, the presented results confirm that FE simulations and analysis based on the simple anisotropic models can capture the observed anisotropic behaviour of cells using the introduced anisotropy metric (D.A.) Further improvements in both model and technique are required for complete characterization of the cell anisotropy.

It is interesting to compare the values of the predicted anisotropic properties required for capturing the observed D.A. to the “effective isotropic” modulus *E*_*eff*_ that would be extracted by ignoring anisotropy and pursuing a traditional Hertzian analysis of the force-indentation curve (in our case *E*_*eff*_ = *E*_1_, the relaxation modulus acquired with the viscoelastic model). The axial modulus *E*_*a*_ could be several orders of magnitude larger, while the transverse modulus *E*_*t*_ was found to be about 50% smaller, respectively compared to the effective isotropic modulus. Thus, for a NIH 3T3 fibroblast from a “cap” group with *E*_1_ ≈ 1.2 kPa and D.A. ≈ 2, the values of *E*_*t*_ ≈ 0.7 kPa, *E*_*a*_ on the order of tens – hundreds kPa and *G*_*a*_ ≈ 0.3–1 kPa are expected.

## Discussion

### Anisotropic indentation behaviour and anisotropic cell stiffness

Anisotropic deformation of adherent cells (mouse myoblasts) was shown previously by stepwise compression at the whole-cell level^50^. Most of the cellular and nuclear deformation was observed perpendicular to the direction of actin filaments present along the long axis of the cell. Our present data confirm the proposed hypothesis that this anisotropic deformation could be attributed to the preferred orientation of actin filaments, more precisely to the orientation of perinuclear actin cap fibers. It can also explain the anisotropic deformation of the nucleus during the indentation^51,52^. Anisotropic deformation pattern was observed here with both actin and membrane staining implying a connection between actin cap fibers and apical membrane, which could be direct or mediated through the cortical actin meshwork^20^.

Most current cell models used for processing of indentation experiments are based on assumptions of isotropic elasticity or viscoelasticity. The anisotropic indentation profile represents a substantial departure from these models. We have found out that the simple three-parameter model (transverse isotropy) can sufficiently explain the observed profiles. The effective modulus measured in the indentation experiment is determined by both anisotropic elastic moduli *E*_*t*_ and *E*_*a*_, but mostly by the transverse moduli. Several-order increase in *E*_*a*_ will cause a relatively small increase in effective moduli (~1–3 times). Thus, indentation experiments alone cannot assess the anisotropy of cells unless some additional modifications in the experiment are used, like the analysis of the indentation profiles along perpendicular directions as it was performed here. The quantitative estimation of all three anisotropic moduli (*E*_*t*_, *E*_*a*_ and *G*_*a*_) requires even more information about the cell structure. However, the numerical analysis with a wide-range variation in the properties of the stress fibers allowed us to obtain the reasonable ranges for *E*_*a*_/*E*_*t*_ and *G*_*a*_/*E*_*t*_ ratios, which could be refined in future with better knowledge about the cell component properties. For anisotropic materials, the dependence of the mechanical parameters from the shape of the indenter was predicted and shown^53^. The use of non-axisymmetric indenters (e.g. pyramidal) could lead to higher values and orientational dependence of measured Young’s modulus (thus, higher variability), which are indeed observed in the AFM experiments on cells^10,54^.

Anisotropic indentation behavior suggests an anisotropic deformability and stiffness of the cells which can be confirmed with FE simulations. Therefore, the cells are stiffer in the direction along the stress fibers and can resist higher forces and withstand higher extensional loads. This is supported by the fact that actin cap fibers usually align with the main cell axis and the direction of the cell movement, which was also observed in the 3D environment^55^. Anisotropic properties could help cells to withstand large magnitude oscillations in diameter of blood vessels (8–10% for human aorta under physiological conditions)^56^. Other examples include the formation and orientation of stress fibers in endothelial cells under the action of the fluid flow^57^ as well as in airway smooth muscle cells under shear stress^58^, which are accompanied by the stiffening of these cells^59^. The anisotropic stiffness in smooth muscle cells was shown for the shear modulus measured with magnetic twisting cytometry^48,58^, where the force is applied in the planar direction to the magnetic bead attached to the apical cell surface. The shear modulus transverse to the long axis of the cell was less than that parallel to the long axis and disruption of the actin cytoskeleton decreased the anisotropy^48^. Anisotropic rheology of cytoplasm was also shown in vascular endothelial cells after application of laminar shear stresses^60^: the direction more compliant to shear deformation gradually aligned parallel to the flow. As suggested by the authors, while the thick stress fibers in the apical plane aligned with flow direction, the rest of the cytoskeletal network in cytoplasm was sparser and therefore more compliant to the shear deformation. Altogether, these findings indicate that mechanical anisotropy is crucial for cells that are exposed to physiological forces especially along one axis, and that actin stress fibers play a major role in this phenomenon.

### Relationship between cytoskeletal organization and mechanical properties

Several conclusions about the relationship between cytoskeletal organization and mechanical properties can be made based on our results presented here. It is known that individual stress fibers are actively tensioned by the action of myosin motors and function as viscoelastic cables that structurally reinforce the cytoskeleton^61^. Thus, tension in perinuclear cap fibers, which like other stress fibers are anchored to the cell substratum and known to be highly contractile^37^, will lead to the compressive force acting on the cell nucleus, microtubules and other intracellular elements underneath these stress fibers. The resulting force balance will determine both the cell shape and apparent cell stiffness. In that sense, the whole cellular structure functions as a pre-stressed scaffold, as described in the tensegrity model^62,63^. In some way, perinuclear stress fibers could be viewed as ultimate manifestation of the contractile prestress within the cell. Cell type, mechanical and biochemical properties of the substrate, and interplay between different cytoskeletal networks all play a role in the resulting force balance, cellular morphology and mechanical properties^43,64–70^. Here, the measurements were performed over the central region of the cell and thus over the nucleus. Yet, we could not expect such a huge difference in stiffness (3x) of the cell with/without the perinuclear cap if the nucleus were to dominate the cellular response, thus confirming the key role of cytoskeletal organization in these measurements.

Although the presence of stress fibers has been correlated with higher cell stiffness in previous studies^1,4,31–34^, the role of different types of stress fibers has not been extensively analysed so far. We assume that the perinuclear actin cap fibers are the major contributor in cell mechanics measured by indentation technique due to high tension^37,42^ and apical location. Other types of stress fibers^39^ are located close to the basal surface (ventral stress fibers) or in special cell regions (dorsal stress fibers and transverse arcs in lamella) and are therefore expected to affect the indentation process to a lesser extent. The actin cortex, a thin network of F-actin and myosin motors that lies under the plasma membrane, plays a major role in cell mechanics when stress fibers are absent. The isotropic tension in the cortical actin will lead to isotropic indentation profile, like seen here in MDA-MB-231 cells and in other cancerous cells^71^.

**Figure 7:**
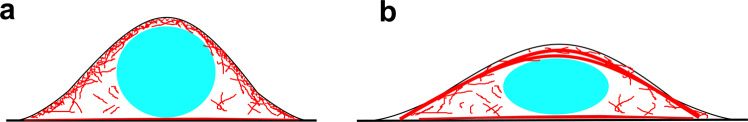
Phenotypes of soft and stiff cells. (**a**) Soft cells could be characterized by higher height, low level of spreading and more viscous and mostly isotropic behaviour. (**b**) Stiff cells have well-developed apical stress fibers, flattened nucleus, they are highly spread and demonstrate more elastic and highly anisotropic mechanical properties.

Therefore, soft and stiff mechanical phenotypes of cells can be described based on indentation experiments combined with imaging of subcellular structures (Fig. 7). Stiff cells, like the majority of the NIH 3T3 fibroblasts, have a well-developed actin cap, low height, flattened nucleus, low power-law exponent (more elastic behaviour) and an anisotropic mechanical response. Soft cells on the other hand, like MDA-MB-231 cells, lack actin cap stress fibers, have larger height, less flattened nucleus, high power-law exponent (more viscous behaviour) and an isotropic mechanical response. This observation agrees well with previous findings. Soft cancer cells lack stress fibers^35,36,72^. A positive correlation was found between the density of stress fibers and cell stiffness^4,31–33^, while cell stiffness and cell height are inversely correlated^34,64^. Furthermore, disruption of stress fibers or inhibition of myosin motors (and thereby reduction of tension) lead to cell softening^1,64,73^. Lastly, the shape of nucleus is controlled by stress fibers, which can lead to its anisotropic deformation^51,52,74,75^.

## Conclusions

In this work, we showed anisotropy in mechanical behaviour of the living cells by direct observation of the local geometry of the membrane deformation during indentation with the integrated AFM and SDC microscopes. Non-axisymmetric indentation profiles were caused by the presence of the perinuclear actin cap stress fibers in NIH 3T3 fibroblasts, which also lead to higher cell stiffness and lower height as expected from the high tension in these fibers. Accordingly, axisymmetric indentation profiles were found in softer cancer MDA-MB-231 cells as well as in NIH 3T3 fibroblasts without any actin cap stress fibers (both naturally occurring and after Latrunculin A treatment). Finite element simulation on transversely isotropic material with just 3 independent parameters allowed capturing the observed non-axisymmetric deformation and estimating the difference in axial and transverse material properties. Our study provides a way to identify the presence of anisotropic mechanical properties in cells by combining AFM indentation with live cell fluorescent stains and SDC microscopy, suggests modelling guidelines to recover their estimates and help predict them based on the cytoskeleton structure. Further studies will shed more light on implications of anisotropic cell mechanical behaviour in mechanical measurements and the role it plays in cell biology.

## Materials and Methods

### Cells

NIH 3T3 and MDA-MB-231 cell lines were maintained in Dulbecco’s modified Eagle’s medium (DMEM) containing 10% FBS and 1% antibiotic/antimycotic solution (Invitrogen, Carlsbad, CA) in a humidified 5% CO_2_ atmosphere at 37 °C. Prior to AFM experiments, cells were re-plated onto 50 mm glass-bottom cell culture dishes (FluoroDish, World Precision Instruments, Sarasota, FL) coated with 30 μg/mL fibronectin for 30 min (Sigma-Aldrich, St. Louis, MO) and grown for an additional period of 1−2 days to a final confluency of ~60–70%. F-actin depolymerizing drug Latrunculin A (Invitrogen, Carlsbad, CA) was applied at a concentration of 100 nM from the DMSO stock solution.

### AFM measurements

AFM measurements were performed using a commercial MFP-3D-Bio AFM (Asylum Research, an Oxford Instruments Company, Santa Barbara, CA) mounted on an IX-71 inverted optical microscope (Olympus, Tokyo, Japan) and integrated with an SDC microscope Andor Revolution XD (Andor Technology, South Windsor, CT). The AFM is equipped with a heated stage and the temperature was kept constant at 30 °C to decrease medium evaporation rate during the experiments (in our preliminary experiments and previous study^31^ the cell mechanical properties was shown to be unchanged in 30–37 °C range). Rectangular AFM cantilevers CSC38 (MikroMasch, Wilsonville, OR) were modified with 5 μm diameter silicon dioxide beads (Microspheres-Nanospheres, Cold Spring, NY) with or without green fluorescence. The bead was glued to the end of the cantilever using UV-curable glue (Optical Adhesive No. 71, Norland Products, Cranbury, NJ). The exact radius of the probe was calculated from AFM images acquired after scanning the test grating TGT01 (MikroMasch, Wilsonville, OR).

The typical spring constant of cantilevers was 0.03–0.05 N/m. The accurate value was determined with laser Doppler vibrometry (Polytec MSA-400 Micro System Analyzer from Polytec GmbH, Waldbronn, Germany), from thermal vibrations in air using the equipartition theorem^76^. Before and after measurements, the relationship between the photodiode signal and cantilever deflection (sensitivity factor S) was calibrated by recording several force curves at a bare region of the glass coverslip and averaging its slope.

For mechanical characterization, the *F-Z* curves were taken above the centre of the cell at 2 μm/s piezo displacement speed along the Z axis. The force set point (~1.2 nN for NIH 3T3 and ~0.8 nN for MDA-MB-231 cells) was chosen individually for all samples to obtain the maximum indentation depth around 500 nm and to reduce the substrate effect^7^. The cell height was determined as a difference between the contact point positions in the *F-Z* curve taken above the bare substrate region next to the cell and in the *F-Z* curve taken above the cell.

Initially, *F-Z* curves were analysed assuming isotropy of the cell mechanical response. Later, finite element analysis (described below) is used to examine the role of local mechanical anisotropy on the observed membrane deformation. Accordingly, the numerical processing of the *F-Z* curves was performed with MATLAB (The MathWorks, Natick, MA) using our previous approach^29^ utilizing Ting’s model^77^:

1 $$F(t,\delta (t))=\{\begin{array}{c}\frac{4\sqrt{R}}{3(1-{\nu }^{2})}{\int }_{0}^{t}E(t-\xi )\frac{\partial {\delta }^{\frac{3}{2}}}{\partial \xi }d\xi ,\,0\le t\le {t}_{m}\\ \frac{4\sqrt{R}}{3(1-{\nu }^{2})}{\int }_{0}^{{t}_{1}(t)}E(t-\xi )\frac{\partial {\delta }^{\frac{3}{2}}}{\partial \xi }d\xi ,\,t\ge {t}_{m}\end{array},$$

2 $${\int }_{{t}_{1}(t)}^{t}E(t-\xi )\frac{\partial \delta }{\partial \xi }d\xi =0,$$

where *F* is the force acting on the cantilever tip; *δ* is the indentation depth; *t* is the time initiated at the contact (*t*_*m*_ is the duration of approach phase); *t*_1_ is the auxiliary function determined by the Eq. 2; *ξ* is the dummy time variable required for the integration; *v* is the Poisson’s ratio of the sample (assumed to be time-independent and equal to 0.5); *R* is the radius of the indenter. *E*(*t*) is the Young’s relaxation modulus for the power-law rheology (PLR) model^29,30,77^:

3 $$E(t)=\,{E}_{1}{t}^{-\alpha },$$

where *E*_1_ is the relaxation modulus at *t* = 1 s (scale factor of the relaxation modulus) and *α* is the power law exponent. A larger *α* value means larger relaxation; materials exhibit a solid-like behaviour at *α* = 0, and a fluid-like behaviour at *α* = 1. Young’s modulus with assumptions of the Hertz’s theory *E*_*Hertz*_ (“apparent”) was also calculated from the approach part of the force curves^78^. It was generally close to the *E*_1_ value since indentation times were on the order of 1 s, yet the *E*_1_ parameter is less dependent from indentation/time rate and thus is presented in the results. The properties calculated from 3 force curves were averaged to obtain the data for the single cell. N = 80 represents the number of cells measured per condition from at least 3 independent experiments (7–30 cells per experiment). The examples of the experimental force curves with the PLR model fit are shown in Fig. S11.

### Spinning disk confocal fluorescence imaging

SiR-actin and SiR-tubulin^79^ were used according to manufacturer’s protocol (Cytoskeleton, Inc., Denver, CO). Briefly, the cells were incubated with 200 nM SiR-dye and 10 µM verapamil (broad spectrum efflux pump inhibitor improving the staining efficiency) diluted in regular growth media overnight. Alternatively, the GFP-actin baculovirus expression vector (Cell-Light^TM^ Actin-GFP BacMam 2.0, Invitrogen) was transfected into the cells according to the manufacturer’s protocol to visualize actin cytoskeleton (overnight incubation). The cells were additionally stained with CellMask Orange plasma membrane staining solution (5 mg/ml; Life Technology) for 10 min when noted in the text. Then, after several washing steps with PBS, visualization and AFM indentation experiments were performed in normal cell growth medium with 20 mM HEPES.

Additionally, the F-actin was visualized in the fixed cells. For that, the cells were fixed with 4% formaldehyde in PBS for 10 min, permeabilized with 0.1% Triton X-100 for 10 min, blocked with 1% bovine serum albumin for 10 min and stained with Alexa Fluor 488 phalloidin (Life Technology).

The fluorescent images were acquired by a spinning-disk confocal microscope Andor Revolution XD with 100x N.A. 1.4 oil immersion objective on the Olympus IX-71 inverted optical microscope. The details about SDC imaging and simultaneous imaging and AFM indentation are given in the Supplementary Information, Sections A and B.

### Finite element analysis

All finite element (FE) simulations were conducted in the commercial FE package Abaqus Standard v. 6.16 (Simulia Corp., Providence, RI). A three-dimensional model representing the indentation of a cube (20 × 20 × 20 µm^3^) made from transversely isotropic material with a rigid sphere (radius 1 µm) was designed. The maximum indentation of 0.5 µm was applied using a displacement-controlled simulation. From the FE simulations data, surface displacement profiles and force vs indentation curves were extracted and analysed. The details about FE simulations are provided in the Supplementary Information, Section E.

### Statistical analysis

Statistical analysis was performed using OriginPro 2016 software (OriginLab Corporation, Northampton, MA). A non-parametric Mann–Whitney U test was used to determine the statistically significant differences between the groups. Since most of the data were not normally distributed (close to log-normal), results are presented both as mean ± standard deviation and median ± median absolute deviation. The percentiles in the box-and-whisker plots are 10%, 25%, 50%, 75% and 90%, the dots are values outside this range.

## Supplementary information

Supplementary Information

Supplementary Movie S1

Supplementary Movie S2

Supplementary Movie S3

## Data Availability

The datasets generated during and/or analysed during the current study are available from the corresponding author on reasonable request.
