# Author Correction: Anisotropy vs isotropy in living cell indentation with AFM

**DOI:** 10.1038/s41598-022-07498-5

**Published:** 2022-03-08

**Authors:** Yuri M. Efremov, Mirian Velay-Lizancos, Cory J. Weaver, Ahmad I. Athamneh, Pablo D. Zavattieri, Daniel M. Suter, Arvind Raman

**Affiliations:** 1 School of Mechanical Engineering, Purdue University, West Lafayette, IN USA; 2 Birck Nanotechnology Center, Purdue University, West Lafayette, IN USA; 3 Lyles School of Civil Engineering, Purdue University, West Lafayette, IN USA; 4 Department of Biological Sciences, Purdue University, West Lafayette, IN USA; 5 Bindley Bioscience Center, Purdue University, West Lafayette, IN USA; 6 Purdue Institute for Integrative Neuroscience, West Lafayette, IN USA; 7 Present Address: Department of Biological Sciences, University of South Carolina, Jones PSC Building, 712 Main Street, Room 517, Columbia, SC 29208 USA

Correstion to: *Scientific Reports* https://doi.org/10.1038/s41598-019-42077-1, published online 08 April 2019

The original version of this Article contained an error in the Discussion, under the subheading ‘Relationship between cytoskeletal organization and mechanical properties’,

 “A positive correlation was found between the density of stress fibers and cell stiffness^4,31,32,33^ and between cell stiffness and cell height^34,64^.”

now reads:

“A positive correlation was found between the density of stress fibers and cell stiffness^4,31,32,33^, while cell stiffness and cell height are inversely correlated^34,64^.”

The original Article has been corrected.

