# Supplementary material for: Anisotropy vs isotropy in living cell indentation with AFM
Source: Sci Rep. 2019 Apr 8;9:5757. doi: 10.1038/s41598-019-42077-1 (PMC6453879; doi:10.1038/s41598-019-42077-1)
Supplement: Supplementary file 1 — Supplementary Information [file 41598_2019_42077_MOESM1_ESM.pdf]

Supplementary Information for

Anisotropy vs isotropy in living cell indentation with AFM

Yuri M. Efremov, Mirian Velay-Lizancos, Cory J. Weaver, Ahmad I. Athamneh, Pablo D. Zavattieri, Daniel M. Suter, and Arvind Raman

Corresponding authors: Arvind Raman, Daniel M. Suter

Email: raman@purdue.edu; dsuter@purdue.edu

The sections of this document are:

a. Spinning disk confocal fluorescence imaging.
b. Simultaneous SDC imaging and AFM indentation
c. Effect of live-cell stains on cell properties
d. Transversely isotropic model
e. Finite element modeling of the indentation experiments
f. Chamis model for the description of cell anisotropy
g. Effect of anisotropy on the force curves

Supplementary Figures S1 to S11

Supplementary Tables S1 to S4

References for SI reference citations

Captions for movies S1 to S3

Other supplementary materials for this manuscript include the following:

Movies S1 to S3

a. Spinning disk confocal fluorescence imaging. The SDC component was mechanically isolated from the microscope and supported by a separate optical table (CSU-X1 Spinning Disk Unit, Yokogawa, Tokyo, Japan). In this way, mechanical vibrations from the spinning disc did not cause any disturbances during the AFM operations. Significant noises were caused by the vertical movement of the objective during optical sectioning, especially for high-aperture immersion objectives. However,

they did not affect the performed measurements since optical sectioning was done when cantilever was retracted from the surface, or during the dwell phase of the force curve (see below, protocol 1, and Fig. S11B), and force curves for mechanical analysis were acquired before the optical sectioning. In a previous study[1], the signal coupling was observed between the epifluorescence excitation light and the cantilever deflection. Here, such coupling was also seen at the high laser intensities, but at the low laser intensities selected for the living cell observation, it was very weak and close to the noise level (Fig. S11C).

Fluorescent microspheres, CellMask, and SiR-dyes were excited by 488 nm, 561 nm, and 640 nm lasers, respectively. The emission signals were separated by 525/30 nm, 607/36 nm, and 685/40 nm filters and recorded by iXon 888BV EMCCD camera (Andor Technology, *South Windsor, CT).* The fluorescent images were analyzed by either Andor IQ software or Fiji (NIH, Bethesda, MD). The orthogonal sections (side views and cross-sections) were reconstructed from the z-stacks of the optical section images using Fiji software. The spacing between the optical sections was chosen based on the Nyquist criterion (0.21 μm for the used setup).

Refractive index (RI) mismatch between the immersion fluid (RI=1.518, Olympus, Japan) and the sample (for aqueous medium, RI is close to 1.33) introduces a decrease in intensity and a shift of the objective focus, thus accurate calibration of the axial distances in confocal microscopy is generally required. We performed this calibration in a preliminary study by placing the AFM probe at a certain distance from the surface (1, 3, 5, 7 μm) using AFM piezo. Then confocal Z-stacks were acquired using SDC from which these distances were calculated as well. By comparison of the distances from the AFM and confocal data, the correction factor 0.88 was obtained for latter which agrees with the theoretical prediction from the previous work[2]. As an additional check, the height of the stained cells calculated using AFM (by the difference in contact points of F-Z curves over the surface and the top of a cell) and from the confocal images was very similar (within 5%).

Synchronization between microscopes was achieved with a TTL trigger signal from AFM to SDC at the beginning of the indentation experiment. The imaging parameters were adjusted in preliminary experiments to decrease the acquisition time and still preserve high signal-to-noise ratio and low phototoxicity. Phototoxicity was observed at high laser intensities as indicated by plasma membrane blebbing, cell detachment, and cytoskeleton disassembly.

To outline the cell membrane or F-actin layer profile in the vertical cross-sections we utilized a method from[3,4]. For each vertical line (x position), the fluorescence peak position was determined by fitting several points around the pixel with maximum intensity with a Gaussian function. The peak of the Gaussian was taken to be the membrane (or F-actin layer) position at x. The width of peaks was comparable to the resolution of the microscope, indicating that the thicknesses of the plasma membrane and F-actin layer are below the resolution limit. The surface displacement profile and indentation depth were found by subtracting the cell profile during indentation from the cell profile prior to indentation. Cell height was measured based on plasma membrane or F-actin staining, since they provided similar results, and both types of fluorescent data were in agreement with the AFM height data.

Classification of cells into three different groups based on the perinuclear actin cap structure was adapted from previous studies[5–7]. The cells with well-developed perinuclear actin cap stress fibers, less-developed fibers (low thickness and low density), and without detectable fibers were classified as the “cap”, “sparse cap”, and “no cap” group, respectively.

b. Simultaneous SDC imaging and AFM indentation. We used three different experimental approaches to combine SDC imaging with AFM indentation to achieve a better visualization of the cell indentation process (Fig. S3). The standard approach used previously (referred to here as the “protocol 1”) is to obtain full z-stack of optical slices for selected cell before and after engagement of the cantilever with prescribed force or indentation depth[3,8,9] (Fig. S3A and Fig. 4 in the main text). In that case, the Z-position of the cantilever is kept constant during the image acquisition (dwell phase of the force curve), but force and indentation depth could change due to cantilever deflection. This approach does not require high temporal resolution, but single z-stack does not provide data on the ongoing viscoelastic relaxation and other force-induced processes in the cell under the engaged cantilever. The relaxation is manifested as decay in the force during the dwell phase of the force curve. A second approach (“protocol 2”), providing the highest temporal resolution, involves the acquisition of a single optical section at selected height during the whole process of cantilever indentation. In this approach, one would expect to see fast rearrangements of cytoskeletal structures but only in the single plane of the view (Fig. S3B, Fig. 3 in the main text, Movies S1-S3). As a compromise, a third approach (“protocol 3”) involves partial Z-stacks, while *F-Z* curves are acquired with the low indentation speed to capture the process (Fig. S3C). Reconstructed cross-sections could

be used to measure the indentation depth (Fig. S4 and S10), which agreed with the AFM data (not shown). The *F-Z* curves were taken at 200 nm/s and 50 nm/s piezo displacement speed along the Z axis for the protocol 3 and 2, respectively. Before implementing any of the described protocols, the mechanical properties of the selected cell were characterized by a set of the force curves taken at 2 μm/s, as described in the Materials and Methods section (with 500 nm maximum indentation depth), and then the force set point for the desired indentation depth was adjusted. Indentation depth was selected to be ~1-1.5 um to obtain well-resolved indentation patterns. For all the protocols, the contact time between the probe and the cell was below 1 min. The examples of the experimental force curves are presented in Fig. S11. After implementation of the protocol, the additional confocal image of the cell was acquired to confirm cell viability and absence of the significant cytoskeletal rearrangements caused by the indentation.

All experiments showed that deformation is reversible at the used indentation parameters (speed, depth and force set-point) (Fig. S9). The force set-point used here was at the level of 1-2 nN, which is comparable with the force cell can generate through a single focal adhesion[10]. Cell viability was preserved and no significant rearrangements of both actin and microtubule cytoskeleton was recorded after the indentation. As shown in a previous study, such rearrangements could be observed when the probe is coated with ECM protein and at longer interaction times[11]. Also, significant remodeling of the microtubule network was found when large probe (50 um diameter) and high forces (20 nN) were applied to the cell[12], but it was not the case here. Membrane patches attached to the probe in some cases, but this did not lead to the cell death or visible damage.

c. Effect of live-cell stains on cell properties. We tested whether the used live-cell imaging stains (probes) affect the mechanical properties of the studied cells as measured by AFM (Fig.1 and Fig S1). Among all stains used, only SiR-actin caused significant cell stiffening. Even the lowest concentrations of the SiR-actin required for imaging (200 nM) led to a significant increase in the relaxation modulus E_1 (~50%, p<0.001; confirmed also in MDA-MB-231 cells, Fig. 1B, Table S1) and decrease in power law exponent α (~20%, p<0.001), meaning solidification of the cell (α=0 for solid material and α=1 for liquid). SiR-tubulin staining, on the other hand, did not lead to significant changes in cell mechanical properties, as well as overnight incubation with the 10 μM verapamil alone. Data for cell

viscoelastic properties are presented in Fig. 1B and Table S1. In agreement with a previous study[13], no effects of SiR probes on cell viability and morphology were noticed. SiR probes are well suitable for live-cell imaging, have excellent brightness and photostability, and more convenient than genetically encoded probes, which require transfection[13]. The SiR-actin probe is based on the jasplakinolide derivative[13], which binds to polymerized actin filaments. Jasplakinolide binding stabilizes actin fibers making them more rigid[14], and is known to promote actin polymerization in the short term[15]. SiR-actin probe could preserve some of its action, which was observed here as increase in cell stiffness (Table 1). We did not notice any effect on viability, morphology, or motility of the cells, which is in agreement with a previous study[13]. SiR-actin did not affect the percentage of the NIH 3T3 with developed actin cap, and stiffening was seen in both fibroblasts and MDA-MB-231 cells, for which stiffness is not based on the stress-fibers, but rather on the cortical actin meshwork. This suggests global cytoskeleton stiffening but not the preferential stiffening of the stress fibers. Due to the effects of SIR-actin on cell stiffness shown here, it should be used with caution in studies where mechanical properties of cells are critical.

SiR-tubulin staining, on the other hand, did not lead to significant changes in cell mechanical properties. SiR-tubulin probe is based on the docetaxel derivative[13], which binds to the microtubules and likely stabilizes them, although SiR-tubulin labeling did not manifest in increased cell stiffness. The dynamic behavior of the microtubules was seen occasionally, probably associated with sliding or disassembly, and was not significantly affected by the indentation process.

Other used fluorescent stains did not significantly affect cell mechanical properties in experimental conditions used here, although some effects from actin-GFP were seen in the previous study[16]. Verapamil, broad spectrum efflux pump inhibitor which was used to improve the SiR-probe labeling efficiency, was shown to affect actin cytoskeleton structure, but at higher concentration and longer incubation times than used here[17].

d. Transversely isotropic model. The compliance matrix S for a transversely isotropic linear elastic material could be written in the following form (assuming the 2–3 plane to be the plane of isotropy):

$$
\begin{bmatrix} \varepsilon_{11} \\ \varepsilon_{22} \\ \varepsilon_{33} \\ \gamma_{12} \\ \gamma_{13} \\ \gamma_{23} \end{bmatrix} = \begin{bmatrix} \frac{1}{E_a} & -\frac{\nu_{ta}}{E_t} & -\frac{\nu_{ta}}{E_t} & 0 & 0 & 0 \\ -\frac{\nu_{at}}{E_a} & \frac{1}{E_t} & -\frac{\nu_t}{E_t} & 0 & 0 & 0 \\ -\frac{\nu_{at}}{E_a} & -\frac{\nu_t}{E_t} & \frac{1}{E_t} & 0 & 0 & 0 \\ 0 & 0 & 0 & \frac{1}{G_a} & 0 & 0 \\ 0 & 0 & 0 & 0 & \frac{1}{G_a} & 0 \\ 0 & 0 & 0 & 0 & 0 & \frac{1}{G_t} \end{bmatrix} \begin{bmatrix} \sigma_{11} \\ \sigma_{22} \\ \sigma_{33} \\ \sigma_{12} \\ \sigma_{13} \\ \sigma_{23} \end{bmatrix}, \tag{1}
$$

where moduli and Poisson's ratios are expressed in terms of "a" for axial (longitudinal) and "t" for transverse (Fig. 6A in the main text). From the seven material parameters E_t, E_a, G_t, G_a, ν_t, ν_{at}, ν_{ta}, only five are mutually independent, while other are related by:

$$
\frac{E_a}{E_t} = \frac{\nu_{at}}{\nu_{ta}}, \tag{2}
$$

$$
G_t = \frac{E_t}{2(1+\nu_t)} \ . \tag{3}
$$

The number of independent parameters reduces further if we apply an assumption about the incompressibility of the material. This requires[18]:

$$
\nu_{at} = 0.5, \tag{4}
$$

$$
\nu_t = 1 - \nu_{ta} = 1 - 0.5\frac{E_t}{E_a} \ . \tag{5}
$$

Thus, only three parameters are independent, which we can select to be E_t, E_a, and G_a. From them, two dimensionless groups were defined: E_a / E_t and G_a / E_t. The anisotropy could arise when $E_a / E_t \neq 1$, or $G_a / G_t \neq 1$, or both. Since the presence of the fibers most likely will lead to an increase in E_a and G_a, in the FE analysis we varied the E_a / E_t from 1 to higher values and G_a / E_t (which is an alternative dimensionless group to G_a / G_t) from 0.33 to higher values (isotropic case is when both $E_a / E_t = 1$ and $G_a / E_t = 0.33$).

e. Finite element modeling of the indentation experiments. The commercial FE package Abaqus Standard v. 6.16 was used for all the simulations reported in this study. A several 3D models were designed. To conduct a general geometry-independent

analysis, the sample was modeled as 20x20x20 μm^3 block and the indenter was a sphere with radius 1 μm. The sphere indented the block up to a maximum penetration of 0.5 μm using a displacement-controlled simulation.

We used a structured mesh composed of 224000 three-dimensional solid elements (C3D8R in Abaqus) 8-node linear bricks, trilinear hexahedral elements, and 238581 nodes; see Figs. S6A and B. The mesh was not uniform, with smaller elements in the indentation area. The characteristic length of the element side in the contact area was 0.1 μm. The indenter was modeled using 38 three-dimensional elements with 3-node (R3D3 in Abaqus), triangular facet and 1043 three-dimensional solid elements with 4-node, bilinear quadrilateral (R3D4 in Abaqus); forming a rigid shell. The mechanical interaction between the block and the indenter was modeled using the general contact surface-to-surface algorithm. The analysis was performed with static step, using the non-linear geometry option. These simulations were used to determine the surface displacement profiles (vertical displacements in the top face of the block) along the XZ and YZ planes (along and normal to the fibers, respectively) and load-displacement curves.

In order to check that the geometry of the sample does not modify significantly the impact of anisotropy on the displacement solution, we performed simulations with block of smaller size (10x10x10 μm^3) and for the ellipsoidal cap representing the cell geometry (Fig. S6C). The dimensions of the ellipsoidal cap were selected to represent the typical dimensions of the cell: base radius a_1 = 30 μm, base radius a_2 = 12 μm (aspect ratio = 2.5 to account for the elongated cell shape), height h = 5 μm, the indenter radius was 2.5 μm and the indentation depth was 1 μm. Only one quarter of the model was simulated by taking the symmetries of the problem into account. For this geometry, the mesh was composed of 25000 10-node quadratic tetrahedron hybrid elements (C3D10H in Abaqus).

For the two considered blocks (large and small), the indentation profiles were fairly close (Fig. S6D, a transversely isotropic material with $E_a / E_t = 1$ and $G_a / E_t = 4$ was employed). These results indicate that even a small block can capture the anisotropy-induced displacement difference. For the ellipsoidal cap geometry, we observed small difference in indentation profiles for the isotropic material. The calculated D.A. was 1.05, which is only 5% deviation from the expected value 1.0 (Fig. S6E). For the transversely isotropic material ($E_a / E_t = 1000$ and $G_a / E_t = 2$) the D.A. value also deviated from the values measured for the block geometry for less than 15%. Thus, we can conclude that even

though sample geometry (and possibly underlying substrate) affects the measured D.A., the effect is reasonably small.

For the computations that follow, we used a block of size 20x20x20 µm^3. All used in simulation ratios E_a / E_t and G_a / E_t are presented in the Table S4 together with the corresponding D.A. and T (relative stiffening, see below) values. $E_t = 100$ and $\nu_{at} = 0.49$ were set constant in all the simulations, other parameters (elastic moduli and Poisson's ratios) were calculated according to Eqs. S2-S5. The range of the ratios was selected based on the D.A. and T values measured for the cells. For very high ratios of E_a / E_t and G_a / G_t severe problems with convergence of simulations were presented and thus these ratios were omitted.

The simulated profiles were processed the same way as the experimental ones, and the degree of anisotropy (D.A.) was calculated for each of them (Fig. 6D in the main text and Table S4). All the simulated force curves (Fig. S6F) were fitted well with the Hertz equation $F = \frac{4}{3} \cdot \frac{E_{eff}}{1-\nu^2} \sqrt{R} \delta^{\frac{3}{2}}$, where parameter E_{eff} represents the "effective" indentation modulus. By normalizing this parameter over its value for the isotropic case (E_{iso}), we obtained the dimensionless ratio $T = E_{eff} / E_{iso}$ (Table S4), representing relative stiffening – a relative change in the material stiffness as measured from the indentation experiment. On the other hand, $T = E_{eff} / E_t$, a ratio of the indentation modulus to the E_t (since E_t was kept constant in the simulations and $E_{iso} = E_t$).

From the FE simulations, we obtained the 2D-surface of the D.A. values as a function of the E_a / E_t and G_a / E_t. We found that G_a / E_t ratio affects surface displacement profiles stronger than E_a / E_t ratio. The largest effect was observed when E_a / E_t and G_a / E_t ratios were increased simultaneously, the resulted D.A. and T values were larger than obtained by simple multiplication of D.A. and T values for the cases when E_a / E_t and G_a / E_t ratios were increased separately. The 2D-surface was interpolated and then intersection with the plane of the D.A.=2.1 (value for the NIH 3T3 fibroblasts with well-developed perinuclear actin cap) was found (Fig. S7A). The intersection determines the line of possible ratios of E_a / E_t and G_a / E_t for the selected D.A. value. However, as seen from the trend in the line on fig. S7B, the selected D.A. value could be obtained by varying only one ratio and keeping another one the same as for the isotropic material. Although

we cannot exactly predict the behavior of the D.A. outside of the range obtained in the simulations, the general trend is that the material can have high E_a / E_t and low G_a / E_t ratio or vice-versa in order to achieve a selected D.A. value. Moreover, the relative stiffening T values were proportional to D.A. values for the simulated materials (Fig. S7C), meaning that one of the values could be estimated based on another one. Thus, if D.A. value is known, E_t could be found based on estimated T and E_{eff} values. Yet, exact values of E_a and G_a could not be determined even when E_t is known due to their concurrent effect: different combinations of E_a / E_t and G_a / E_t can provide very close simulated force curves and displacement profiles (Fig. S7D, E), as expected from the close D.A. and T values.

At this step, we want to obtain a range of physically reasonable values; the highest and the lowest estimates for both ratios which might be expected in cells with actin cap fibers. From the trend line, it follows that higher estimate for G_a / E_t will correspond to the lowest estimate for E_a / E_t and vice versa. To obtain these estimates, we applied the analysis based on the rule of mixtures (Chamis model)[19,20]. Alternatively, if at least one ratio, G_a / E_t or E_a / E_t, could be obtained from the independent measurement, the remaining ratio could be caluclated in a straightforward way from the trend line (Fig. S7B).

f. Chamis model for the description of cell anisotropy. An alternative view on a transversely isotropic material is coming from the theory of fiber-reinforced composite materials [20]. For unidirectional composite materials with long fibers, the mechanical properties could be predicted with the so-called rule of mixture ("*ROM*"). Involved assumptions are: fibers are uniformly distributed throughout the matrix; fiber and matrix behave as linearly elastic materials (matrix is isotropic, but fibers could be anisotropic by itself). The properties of the matrix are E^m, ν^m and the volume fraction is V^m; the properties of fibers are E^f, ν^f and the volume fraction is V^f, $V^f + V^m = 1$. Here we used Chamis model[19,20], which is more advanced than original *ROM* model. It is also one of the most used and trusted model which give a formulation for all five independent elastic properties of the composite:

$$E_a = V^f E_a^f + V^m E^m , \quad (6)$$

$$E_t = \frac{E^m}{1 - \sqrt{V^f}(1 - E^m / E_t^f)} , \quad (7)$$

$$\nu_{at} = V^f \nu_{at}^f + V^m \nu^m , \quad (8)$$

$$G_a = \frac{G^m}{1 - \sqrt{V^f}(1 - G^m / G_a^f)} , \quad (9)$$

$$G_t = \frac{G^m}{1 - \sqrt{V^f}(1 - G^m / G_t^f)} . \quad (10)$$

Since mechanical properties of the stress fibers are not exactly known and will depend on the pre-stress, we can only make a numerical analysis with some relevant range of values. We also assumed that when the cell lacks the apical stress fibers as seen from the SDC images, the measured values corresponded to the matrix (including membrane, cytoplasm, actin cortex and other components, but not the stress fibers). The stress fibers were considered to be stiffer than the matrix[21,22], $10 < E_a^f / E^m < 10^5$, $1 < E_t^f / E^m < 10^4$, $10 < G_a^f / G^m < 10^5$, $1 < G_t^f / G^m < 10^4$; and the volume fraction was varied in the range $0.01 < V^f < 0.6$. As seen from the equations, the Poisson's ratio of fibers does not affect other mechanical parameters, so they were not considered here. Simple numerical analysis (Fig. S8) showed that presence of stiff fibers affects E_a much more than E_t leading to a pronounced increase in E_a / E_t ratio (up to ~60000). The G_a / G_t ratio, on the other hand, is entirely determined by the anisotropy in mechanical properties of fibers itself. Following the equations, even high G_a^f / G_t^f ratios (10^5) transform to small G_a / G_t ratios of the composite material (up to ~5). This analysis confirms the possibility of the high E_a / E_t ratio and smaller G_a / G_t ratio in cells and leads to the following estimations: the lowest estimate for G_a / E_t ratio is 0.33 (no change from the isotropic case) and the highest estimate for E_a / E_t ratio is ≈60000. The highest estimate for G_a / E_t is ≈1.5 ($G_a / G_t \approx 4$) and the lowest estimate for E_a / E_t ratio is ~400 (from Fig. S7B). This estimate is in agreement with the data from the previous study where $G_a / G_t \approx 2$ was reported for the human airway smooth muscle cells[23]. We can expect high E_a / E_t ratios (hundreds and more) in NIH 3T3 fibroblasts if they have a G_a / G_t in the range of 2-4.

g. Effect of anisotropy on the force curves. It could be noted from the Table S4 and Fig. S7C that relative stiffening *T* values are generally proportional to and lower than D.A. values for the simulated materials. Thus, the materials with the higher level of anisotropy

also provides higher indentation modulus. The effective modulus measured in the indentation experiment from the force curves (E_{eff}, which in our measurements was relaxation modulus E_1, but also could be the Young's modulus obtained with the Hertz theory for the purely elastic case) is determined by both anisotropic moduli E_t and E_a, but mostly by the transverse moduli. Several-order increase in E_a will cause relatively small increase in effective moduli (~1-3 times, Fig. S6G). Thus, indentation experiments alone can't assess the anisotropy of cells unless some additional modifications in the experiment are used, like analysis of the indentation profiles performed here. By interpolating T vs D.A. dependence (Fig. S7C), the T value could be established from the experimental D.A. value, and then E_t could be calculated from the effective indentation modulus. For example, in NIH 3T3 fibroblasts with D.A. ≈ 2.1, the $T \approx 2$ is expected meaning that the transverse modulus is twice lower than the E_{eff} measured from the force curve.

An additional observable is needed to calculate both E_a and G_a independently. In a future study, we hope to get this information from additional experiments. It could be a use of asymmetric indenter with indentation before and after 90 degrees indenter rotation[24]; implementation of lateral force microscopy for the measurement of shear modulus in two directions; combination of AFM with other techniques, where forces could be applied to the probe in lateral direction (like optical or magnetic tweezers)[23].

Supplementary Figures

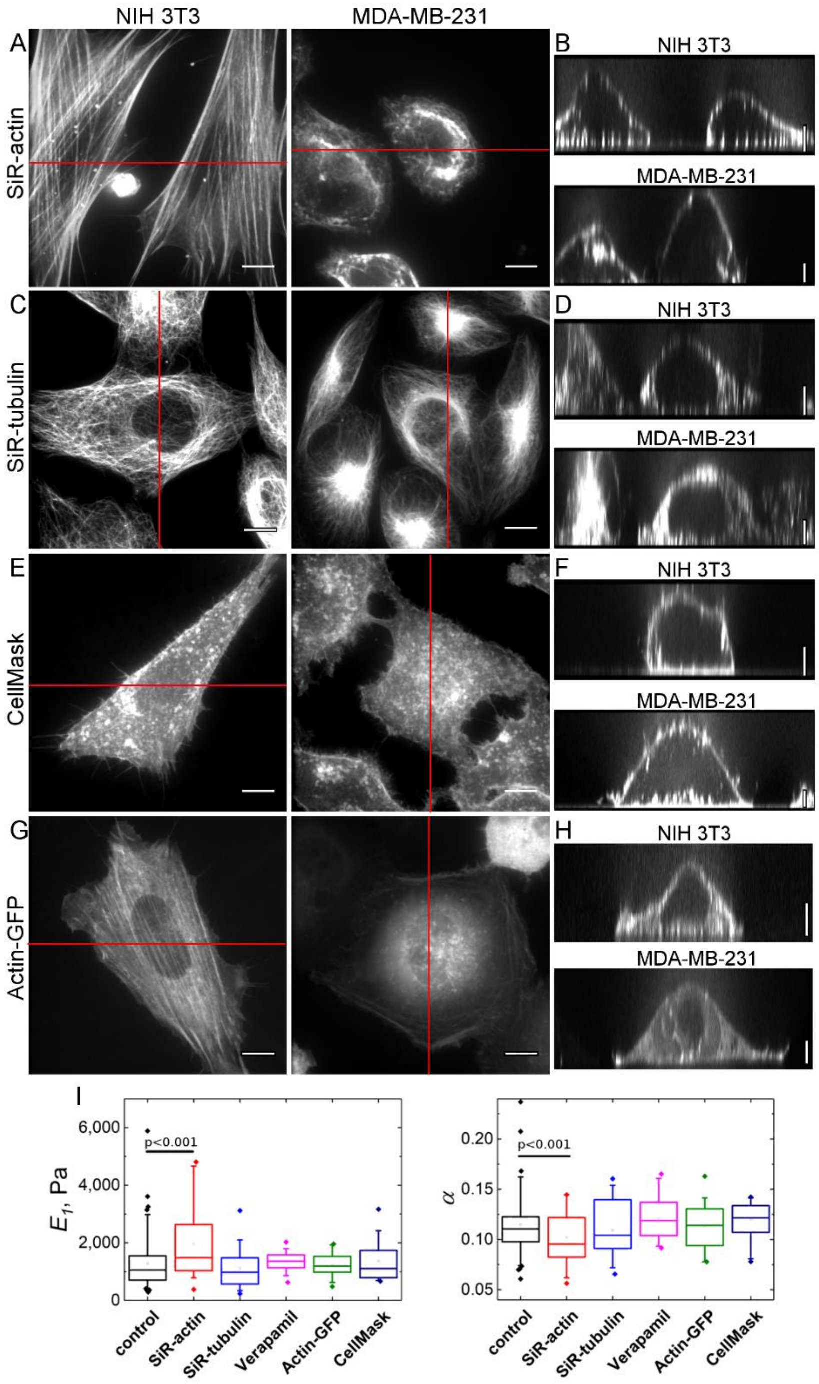

Fig. S1. F-actin (SiR-actin - A, B; Actin-GFP – G, H), microtubules (SiR-tubulin, C, D) and plasma membrane (CellMask Orange - E, F) structure in NIH 3T3 and MDA-MB-231 cells, Z-projections (A, C, E, G) and vertical cross-sections along marked lines (B, D, F, H). Distinctive stress-fibers are present in NIH 3T3 cells both in basal and apical planes, but rarely in MDA-MB-231 cells, where F-actin is mostly found in the cortex. The microtubules were located homogenously throughout the cytoplasm outside the nucleus in both cell types. The plasma membrane staining was used for assessment of the cell shape and indentation profiles. Scale bars 10 μm in the horizontal direction and 2 μm in the vertical direction. (I) Box plots of Young's relaxation modulus scale factor E_1 and power-law exponent α for NIH 3T3 cells after application of different live-cell imaging stains. The differences between all distributions except the one marked are not significant at the p<0.01 level.

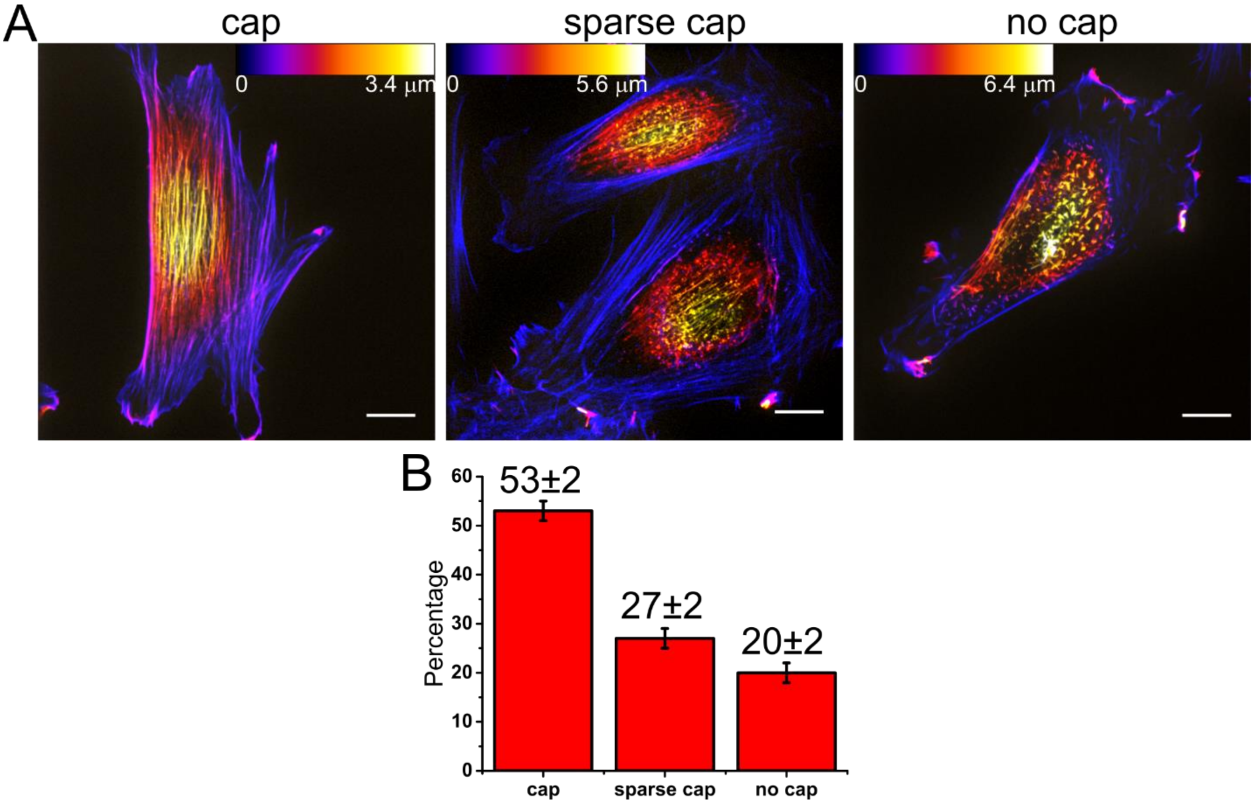

Fig. S2. F-actin cytoskeleton in fixed NIH 3T3 fibroblasts stained with Alexa Fluor 488 phalloidin. (A) Typical distribution of actin in fibroblasts with well-developed actin cap (cap), sparse cap, and with no cap. The height is color coded with respect to the scaling shown in the color scale bars. Scale bars 10 μm. (B) Percentage of cells in different groups measured from 3 independent experiments (748 cells analyzed).

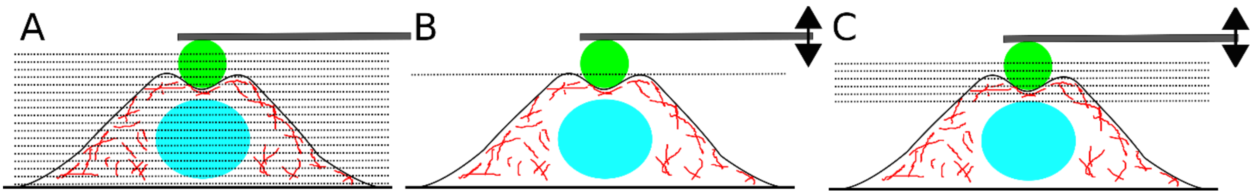

Fig. S3. Schemes of different indentation/imaging protocols used in this study. (A) Full z-stack before the indentation and during the dwell period, Z-coordinate of the cantilever base is kept constant during the imaging (protocol 1). (B) Single optical section during the indentation (protocol 2), the cantilever base is moved with the constant speed. (C) Partial z-stack during the indentation (protocol 3), the cantilever base is moved with the slow constant speed. Horizontal planes represent single optical sections.

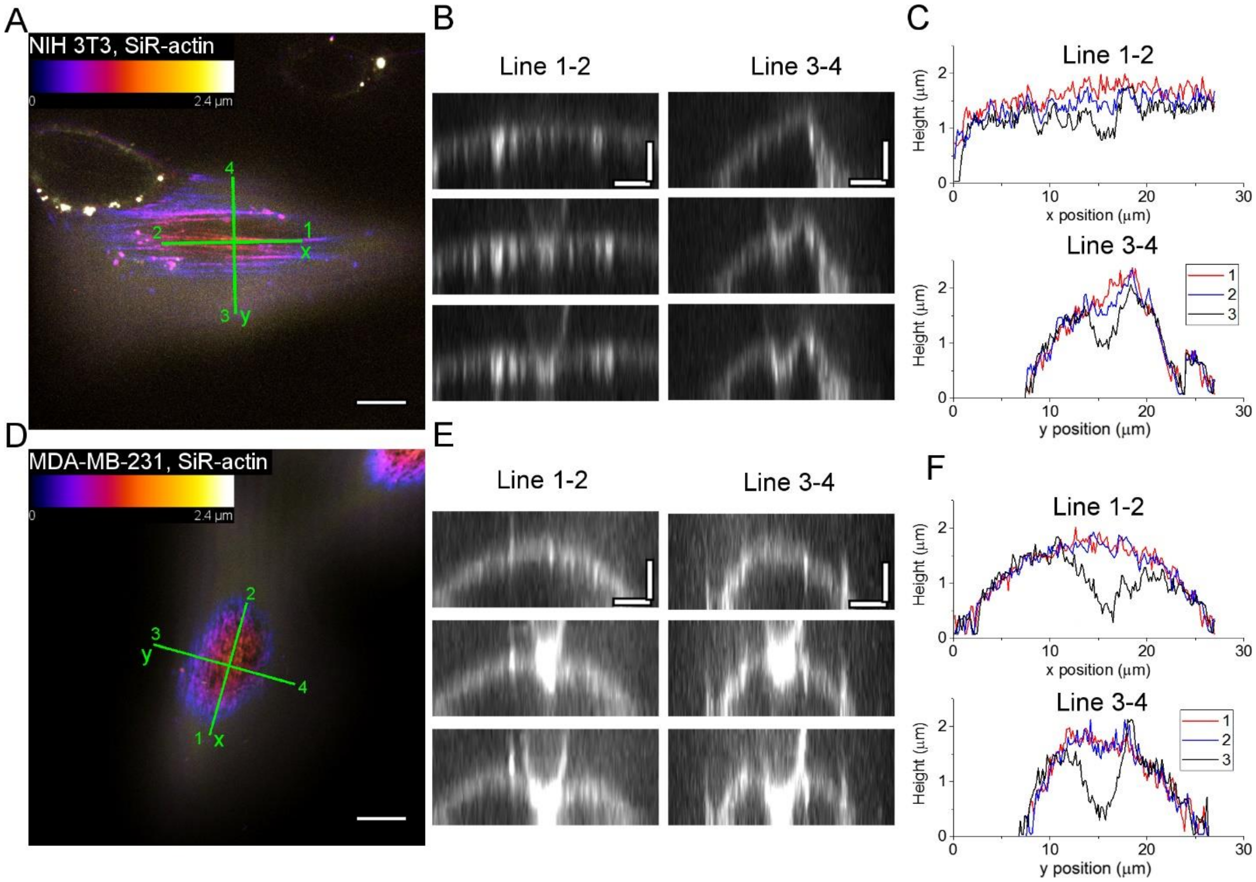

Fig. S4. Anisotropic (non-axisymmetric) and isotropic indentation profiles in NIH 3T3 (A-C) and MDA-MB-231 (D-F) cells, respectively. Partial Z-stack experiment (protocol 3, see Supplementary Information, Section B). SiR-actin (A, D) and CellMask (B, E) staining. (A, D) Color coded z-projections of the SiR-actin staining. (B, E) Reconstructed vertical cross-sections along the marked lines, three time points (top to bottom). (C, F) The calculated position of the membrane. Red line 1 is the first, blue line 2 is the second, and black line 3 is the third time point. Scale bars: 10 μm for the z-projections; 5 μm in the horizontal direction and 1 μm in the vertical direction for the cross-sections. In NIH 3T3 fibroblasts, the cap fibers go deeper as a whole structure during the indentation, while deforming slightly in the area of contact. In MDA-MB-231 cells, a more isotropic deformation pattern was observed.

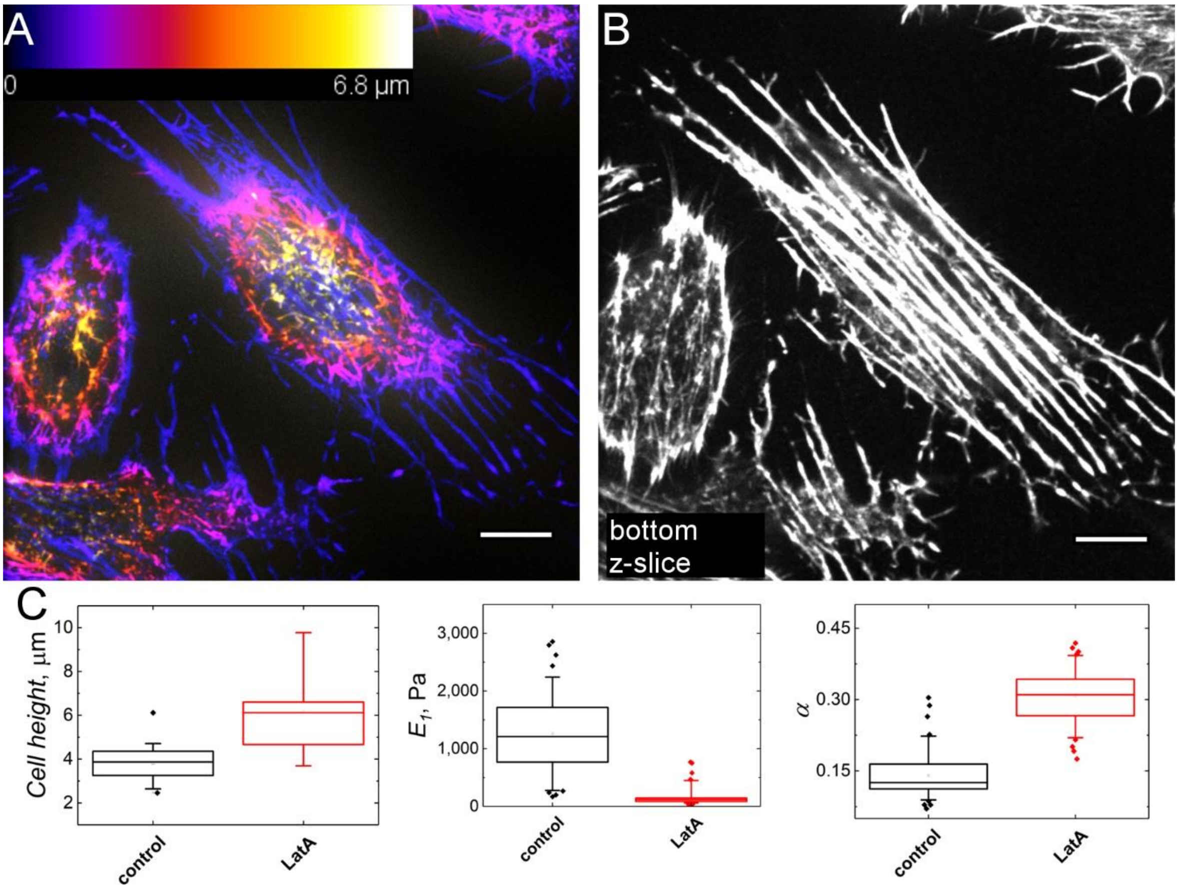

Fig. S5. Disruption of apical cap fibers after Latrunculin A treatment (100 nM, 30 min). (A) Color coded z-projections of the SiR-actin staining showing absence of the apical cap. (B) Bottom z-slice of the stack showing well-preserved ventral stress fibers. (C) Box plots of cell height, Young's relaxation modulus scale factor E_1, and power-law exponent α (Data for cells with SiR-actin staining). The differences between all distributions are significant at the p<0.01 level.

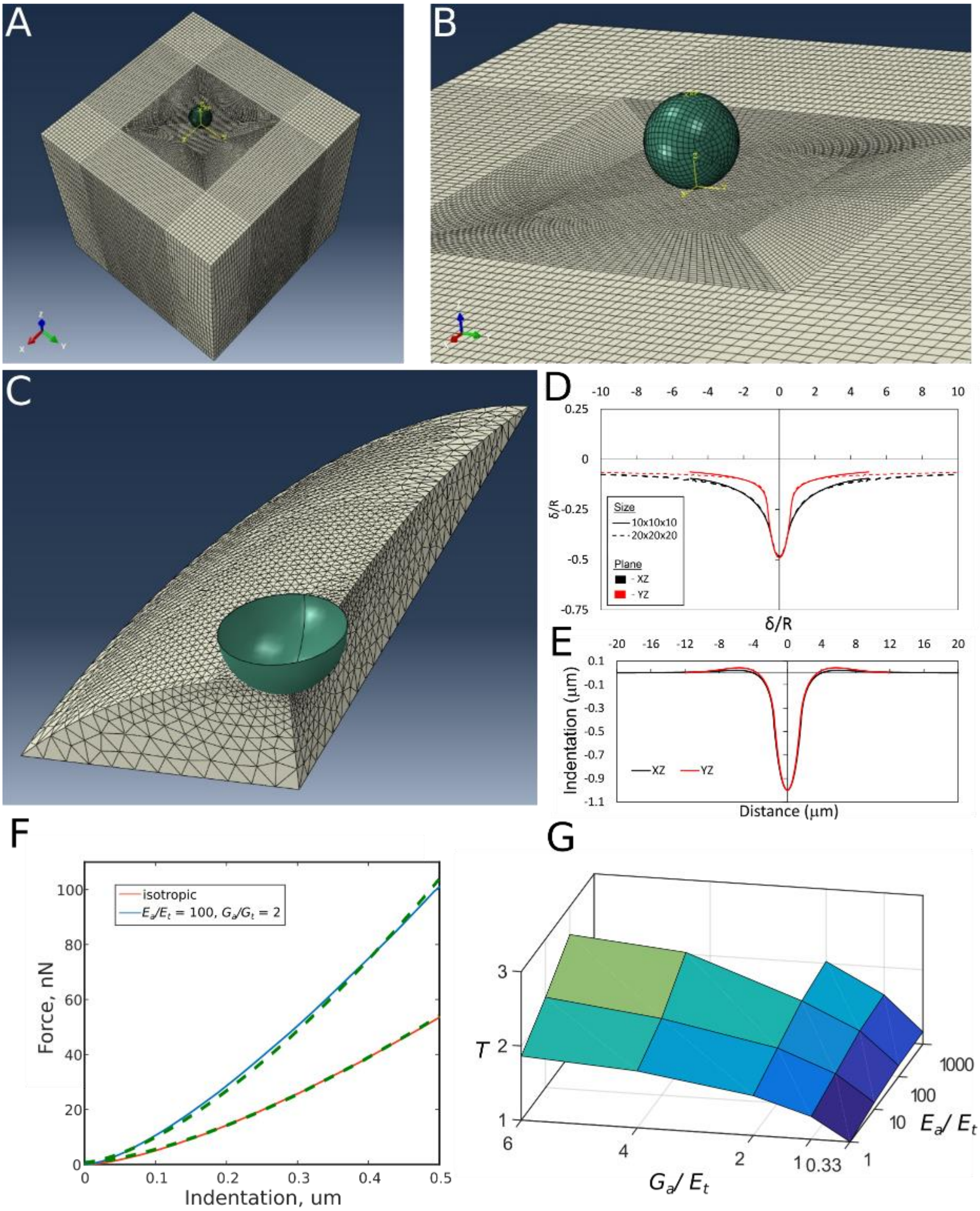

Fig. S6. Additional information for the FEM simulations. 3D geometry used in the simulations, general (A) and close-up (B) view. (C) The ellipsoidal cap representing the cell geometry (only one quarter was used for simulation with the symmetry conditions). (D) Comparison between the indentation profiles obtained with different size of the block. (E) The indentation profile obtained for the ellipsoidal cap geometry. (F) The force curves obtained from the FEM simulations for isotropic and anisotropic material. The fit with the Hertz model is shown with the dashed curves. (G) The surface plot of the relative stiffening T versus G_a / E_t (linear scale) and E_a / E_t (log scale) ratios.

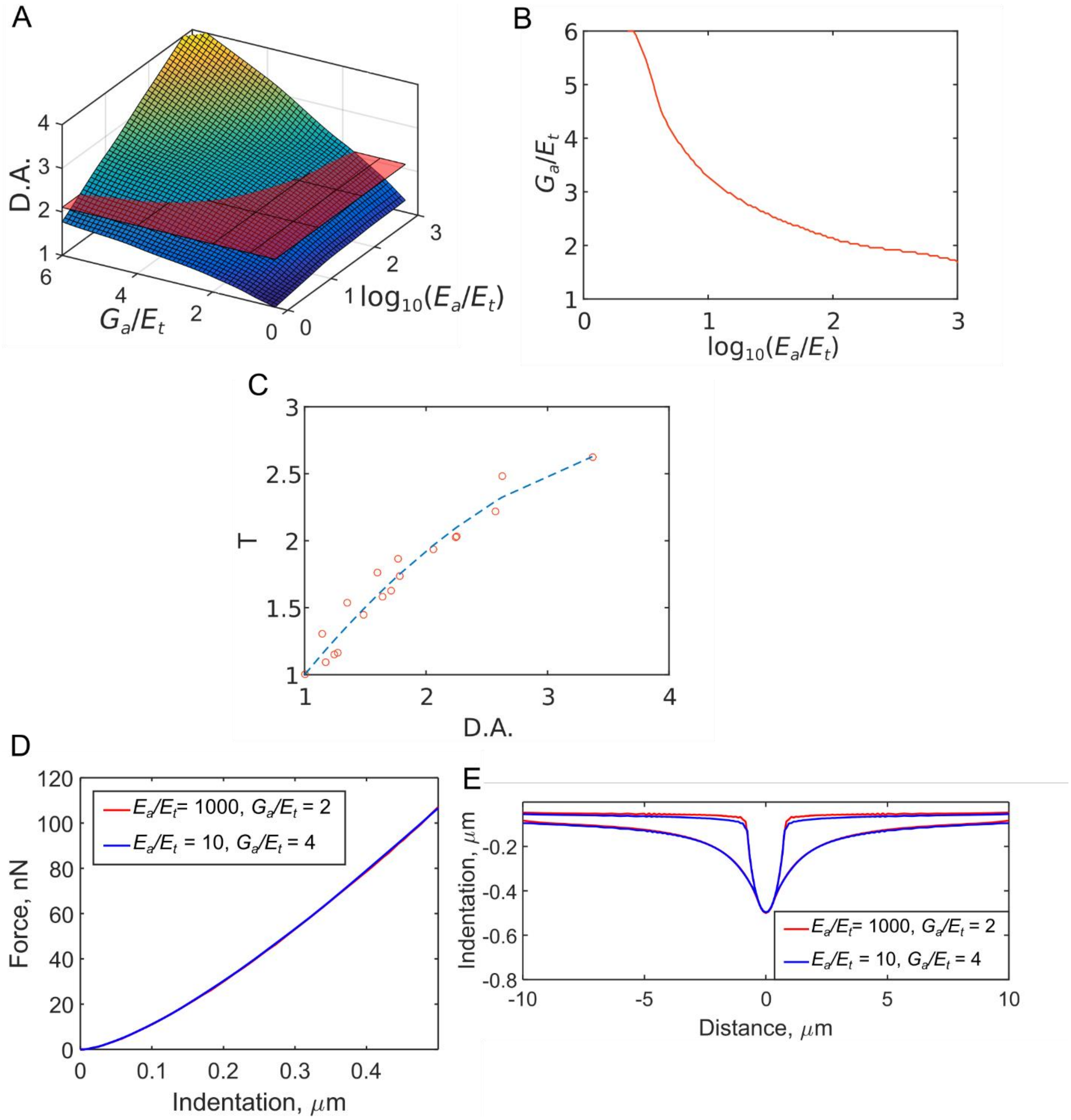

Fig. S7. Additional information for the FEM simulations. (A) The interpolated surface plot of the degree of anisotropy (D.A.) versus G_a / E_t and $\log_{10}(E_a / E_t)$; the red plane marks D.A.=2.1. (B) The line obtained from intersection of the surface and the plane. (C) Relative stiffening (T) values versus D.A. values for the conducted FEM simulations, dashed line represents polynomial fit. (D, E) Different sets of G_a / E_t and E_a / E_t ratios can provide very similar simulated force curves (D) and indentation profiles (E).

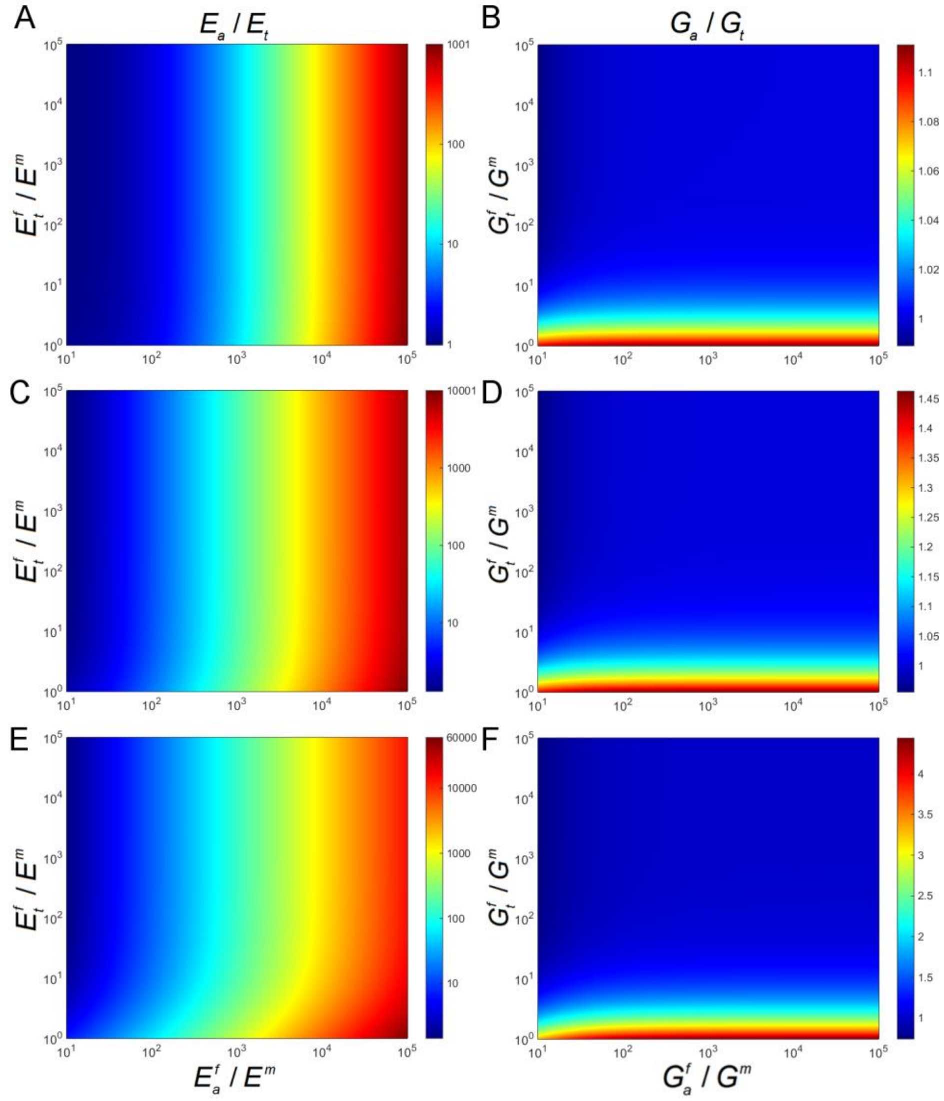

Fig. S8. Numerical predictions of the Chamis model for the different fiber parameters. E_a / E_t (A, C, E) and G_a / G_t ratios (B, D, F), were calculated for the volume fraction of fibers 0.01 (a, b); 0.1 (c, d); 0.6 (e, f). See color scale bars for values.

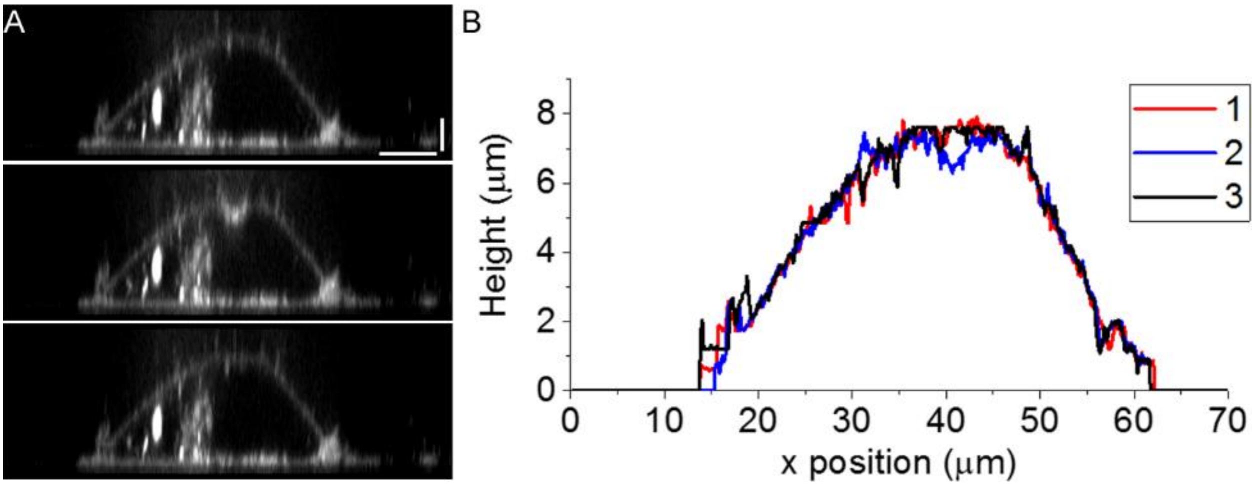

Fig. S9. Reversible indentation of MDA-MB-231 cells. (A) The vertical cross-sections prior to (1), during (2) and after the probe engagement (3, from top to bottom). Plasma membrane was stained with CellMask. (B) The calculated position of the membrane. Scale bar 10 μm in horizontal direction and 2 μm in vertical direction.

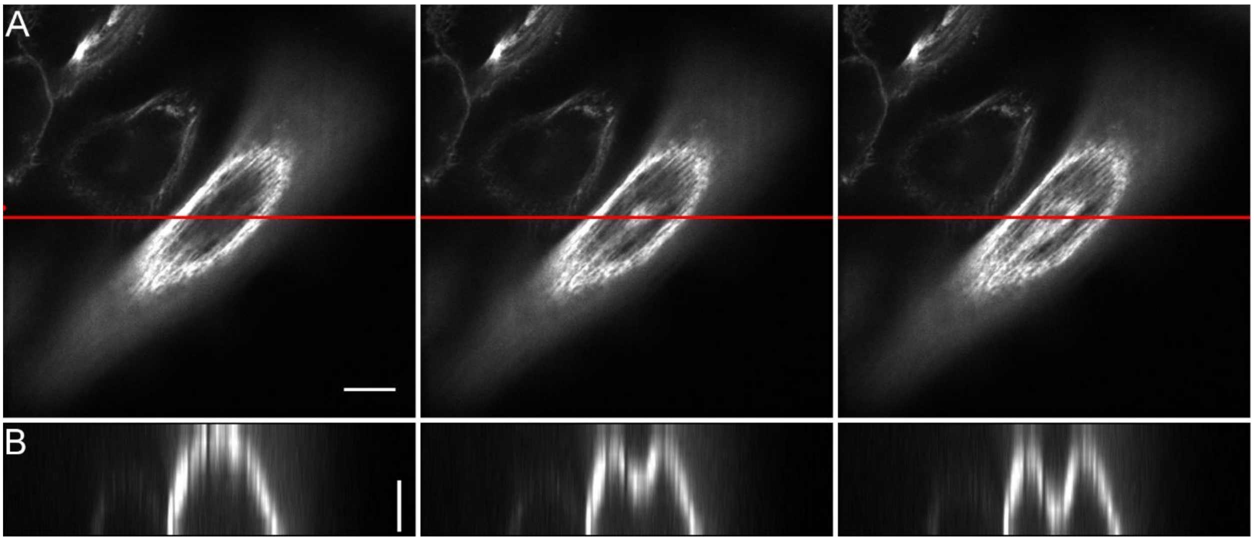

Fig. S10. Indentation of MDA-MB-231 cells labeled with SiR-actin, partial Z-stack experiment,10 Z-planes (protocol 3, see protocol 1, Supplementary Information, Section B). (A) The single optical plane in the middle of the z-stack. (B) Reconstructed vertical cross-section. The bead fluorescence signal is not recorded to decrease acquisition time. The time required to record partial Z-stack is ~3s. Scale bars: 10 μm in the horizontal direction and 1 μm in the vertical direction.

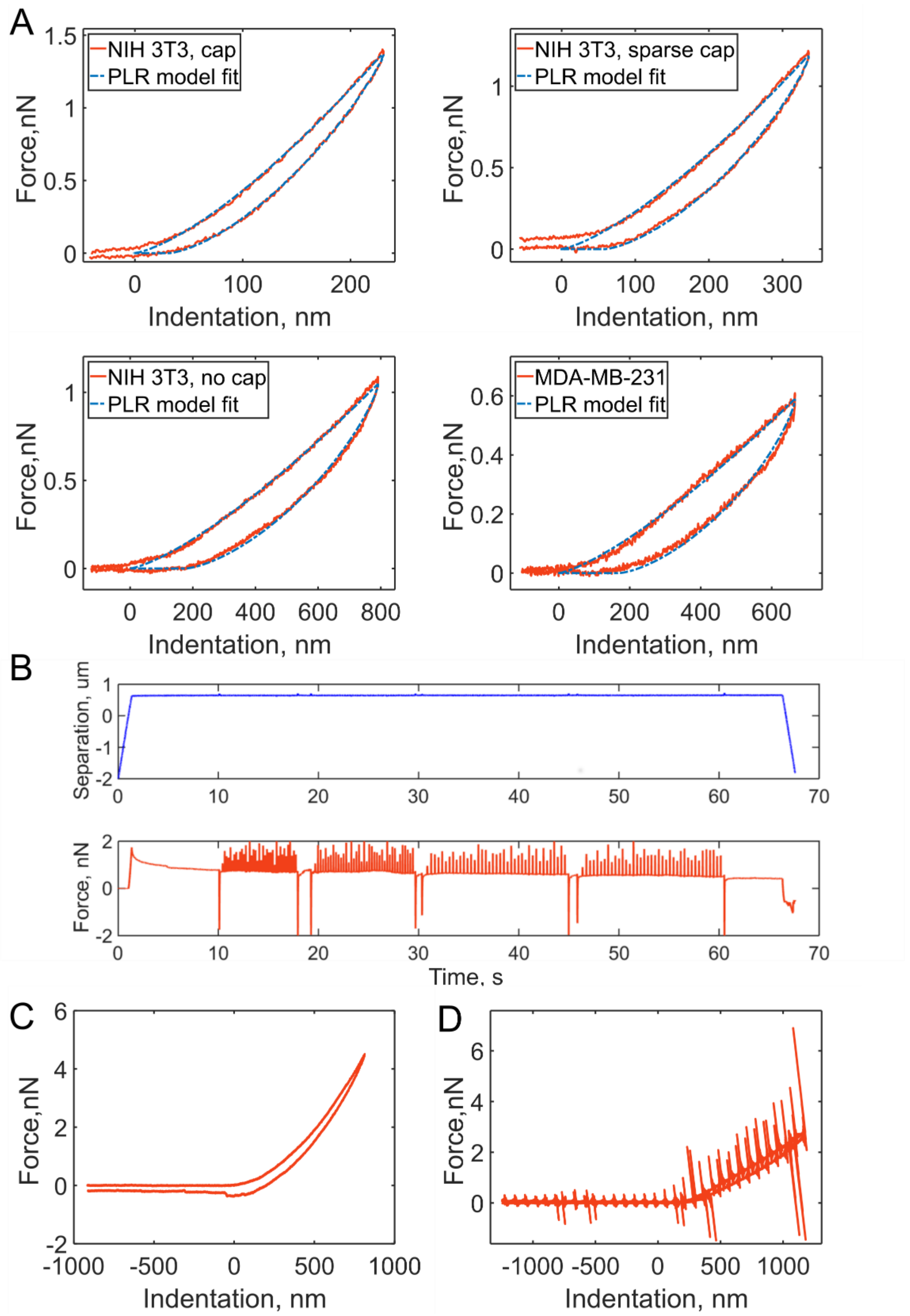

Fig. S11. Examples of the force curves obtained in the experiments. (A) Examples of the force curves which were used for viscoelastic characterization of the cells with the power law rheology model (PLR) obtained on NIH 3T3 cells with a well-developed actin cap, sparse cap, without actin cap, and on MDA-MB-231 cell The fit with PLR model is shown. (B) An example of the force curve obtained by the protocol 1 (the Z-position of the cantilever is kept constant during the image acquisition), the spikes of the deflection signal during the dwell phase are caused by the optical sectioning during the SDC image acquisition. (C) An example of the force curve obtained by the protocol 2 (the acquisition of a single optical section at selected height during the continuous indentation cycle), no signal coupling between the excitation laser and the cantilever deflection was detected. (D) An example of the force curve obtained by the protocol 3 (partial z-stack during the indentation).

Supplementary Tables

Table S1. Viscoelastic properties of cell lines used in this study after application of living cell fluorescent stains. Mean ± s.d. (median ± m.a.d.). SiR probes: 10 μM verapamil + 200 nM SiR-actin or tubulin; 10 μM verapamil alone; Cell-Light^{TM} Actin-GFP BacMam 2.0 overnight transfection; CellMask 1/1000 10 min staining. N is the number of measured cells.

Cell line	E_1 (kPa)	α
NIH 3T3 control (N=90)	1.3 ± 0.9 (1.1 ± 0.4)	0.11 ± 0.03 (0.11 ± 0.01)
+ SiR-actin (N=90)	2.0 ± 1.2 (1.5 ± 0.6)	0.10 ± 0.02 (0.09 ± 0.02)
+ SiR-tubulin (N=26)	1.1 ± 0.7 (1.0 ± 0.4)	0.11 ± 0.03 (0.10 ± 0.02)
Verapamil (N=22)	1.3 ± 0.3 (1.4 ± 0.2)	0.12 ± 0.02 (0.12 ± 0.02)
Actin-GFP (N=30)	1.2 ± 0.4 (1.2 ± 0.3)	0.11 ± 0.02 (0.11 ± 0.02)
CellMask (N=27)	1.4 ± 0.7 (1.1 ± 0.4)	0.12 ± 0.02 (0.12 ± 0.02)
MDA-MB-231 control (N=67)	0.6 ± 0.5 (0.6 ± 0.3)	0.13 ± 0.04 (0.12 ± 0.02)
+ SiR-actin (N=30)	0.9 ± 0.5 (0.9 ± 0.3)	0.12 ± 0.03 (0.11 ± 0.02)
+ SiR-tubulin (N=30)	0.6 ± 0.3 (0.5 ± 0.2)	0.12 ± 0.04 (0.11 ± 0.03)
Verapamil (N=33)	0.6 ± 0.3 (0.6 ± 0.2)	0.14 ± 0.05 (0.14 ± 0.03)
Actin-GFP (N=33)	0.5 ± 0.3 (0.4 ± 0.2)	0.15 ± 0.04 (0.15 ± 0.03)
CellMask (N=33)	0.7 ± 0.5 (0.6 ± 0.3)	0.14 ± 0.05 (0.13 ± 0.03)

Table S2. Viscoelastic properties and cell height of NIH 3T3 cells with different level of perinuclear actin cap development (checked with SiR-actin staining). Mean ± s.d. (median ± m.a.d.). N is the number of measured cells.

Cap level	E_1 (kPa)	α	Height, μm
cap (N=47)	1.9 ± 0.8 (1.7 ± 0.4)	0.11 ± 0.02 (0.11 ± 0.01)	3.7 ± 0.7 (3.7 ± 0.4)
sparse cap (N=31)	1.2 ± 0.6 (1.1 ± 0.2)	0.12 ± 0.03 (0.12 ± 0.02)	4.6 ± 0.8 (4.5 ± 0.6)
no cap (N=20)	0.7 ± 0.4 (0.7 ± 0.3)	0.15 ± 0.04 (0.16 ± 0.03)	5.4 ± 0.9 (5.5 ± 0.6)

Table S3. Viscoelastic properties and cell height of NIH 3T3 cells with different level of perinuclear actin cap development (checked with Cell-Light™ Actin-GFP BacMam 2.0). Mean ± s.d. (median ± m.a.d.). N is the number of measured cells.

Cap level	E_1 (kPa)	α	Height, μm
cap (N=13)	1.2 ± 0.3 (1.1 ± 0.1)	0.11 ± 0.02 (0.11 ± 0.01)	3.7 ± 0.5 (3.7 ± 0.5)
sparse cap (N=9)	0.7 ± 0.2 (0.6 ± 0.1)	0.13 ± 0.04 (0.14 ± 0.02)	4.0 ± 0.4 (4.1 ± 0.3)
no cap (N=3)	0.4 ± 0.1 (0.4 ± 0.1)	0.19 ± 0.04 (0.20 ± 0.03)	4.6 ± 0.5 (4.6 ± 0.6)

Table S4. ***D.A.*** **(degree of anisotropy, upper value in the cell) and *T* (relative stiffening, lower value in the cell) for the performed finite element simulations**

		E_a / E_t				
		**1**	**10**	**100**	**1000**	**10^6**
G_a / E_t	**0.33**	1<br>1	1.17<br>1.09	1.25<br>1.15	1.28<br>1.16	did not converge
	**1**	1.15<br>1.31	1.49<br>1.44	1.64<br>1.58	1.71<br>1.63	-
	**2**	1.35<br>1.54	1.79<br>1.742	2.06<br>1.93	2.24<br>2.03	-
	**4**	1.60<br>1.76	2.25<br>2.03	2.62<br>2.48	did not converge	-
	**6**	1.77<br>1.86	2.57<br>2.21	3.37<br>2.62	did not converge	did not converge

Supplementary movies

Supplementary Movie S1. Deformation of single apical stress fibers in NIH 3T3 fibroblasts causes anisotropic (non-axisymmetric) indentation profile. Single-plane recording experiment (protocol 2, see Methods). F-Actin (left, SiR-actin) and plasma membrane (right, CellMask) staining. The perinuclear actin cap fibers located underneath the bead deformed most, going deeper out of the focal plane. Anisotropic deformation pattern was also observed with membrane staining as a decrease in the dye intensity along the fiber direction and an extension in the perpendicular direction. During this movie, the maximum indentation depth of 800 nm was reached with the force of 5 nN. Scale bar 10 μm.

Supplementary Movie S2. Isotropic indentation profile in MDA-MB-231 cells. Single-plane recording experiment (protocol 2, see Methods). SiR-actin staining. During this movie, the maximum indentation depth of 2000 nm was reached with the force of 3.5 nN. Scale bar 10 μm.

Supplementary Movie S3. Isotropic indentation profile in MDA-MB-231 cells. Single-plane recording experiment (protocol 2, see Methods). CellMask Orange membrane staining. During this movie, the maximum indentation depth of 2000 nm was reached with the force of 3.5 nN. Scale bar 10 μm.

References

1. Cazaux, S. *et al.* Synchronizing atomic force microscopy force mode and fluorescence microscopy in real time for immune cell stimulation and activation studies. *Ultramicroscopy* **160,** 168–181 (2016).
2. Besseling, T. H., Jose, J. & Blaaderen, A. Van. Methods to calibrate and scale axial distances in confocal microscopy as a function of refractive index. *J. Microsc.* **257,** 142–150 (2015).
3. Harris, A. R. & Charras, G. T. Experimental validation of atomic force microscopy-based cell elasticity measurements. *Nanotechnology* **22,** 345102 (2011).
4. Clark, A. G., Dierkes, K. & Paluch, E. K. Monitoring actin cortex thickness in live cells. *Biophys. J.* **105,** 570–80 (2013).
5. Kidoaki, S., Matsuda, T. & Yoshikawa, K. Relationship between apical membrane elasticity and stress fiber organization in fibroblasts analyzed by fluorescence and atomic force microscopy. *Biomech. Model. Mechanobiol.* **5,** 263–272 (2006).
6. Khatau, S. B. *et al.* A perinuclear actin cap regulates nuclear shape. *Proc. Natl. Acad. Sci. U. S. A.* **106,** 19017–19022 (2009).
7. Maninová, M. & Vomastek, T. Dorsal stress fibers, transverse actin arcs and perinuclear actin fibers form an interconnected network that induces nuclear movement in polarizing fibroblasts. *FEBS J.* **283,** 3676–3693 (2016).
8. Vargas-Pinto, R., Gong, H., Vahabikashi, a. & Johnson, M. The Effect of the Endothelial Cell Cortex on Atomic Force Microscopy Measurements. *Biophys. J.* **105,** 300–309 (2013).
9. Melzak, K. a. & Toca-Herrera, J. L. Atomic force microscopy and cells: Indentation profiles around the AFM tip, cell shape changes, and other examples of experimental factors affecting modeling. *Microsc. Res. Tech.* **78,** 626–632 (2015).
10. Balaban, N. Q. *et al.* Force and focal adhesion assembly: a close relationship studied using elastic micropatterned substrates. *Nat. Cell Biol.* **3,** 466–472 (2001).
11. Lim, S.-M., Trzeciakowski, J. P., Sreenivasappa, H., Dangott, L. J. & Trache, A. RhoA-induced cytoskeletal tension controls adaptive cellular remodeling to mechanical signaling. *Integr. Biol.* **4,** 615 (2012).
12. Celik, E., Abdulreda, M. H., Maiguel, D., Li, J. & Moy, V. T. Rearrangement of microtubule network under biochemical and mechanical stimulations. *Methods* **60,** 195–201 (2013).
13. Lukinavičius, G. *et al.* Fluorogenic probes for live-cell imaging of the cytoskeleton. *Nat. Methods* **11,** 731–3 (2014).
14. Visegrády, B., Lorinczy, D., Hild, G., Somogyi, B. & Nyitrai, M. The effect of phalloidin and jasplakinolide on the flexibility and thermal stability of actin filaments. *FEBS Lett.* **565,** 163–166 (2004).
15. Kronlage, C., Schäfer-Herte, M., Böning, D., Oberleithner, H. & Fels, J. Feeling for Filaments: Quantification of the Cortical Actin Web in Live Vascular Endothelium. *Biophys. J.* **109,** 687–698 (2015).
16. Sliogeryte, K. *et al.* Differential effects of LifeAct-GFP and actin-GFP on cell mechanics assessed using micropipette aspiration. *J. Biomech.* **49,** 310–317 (2016).

17. Boggio, R. F., Freitas, V. M., Cassiola, F. M., Urabayashi, M. & MacHado-Santelli, G. M. Effect of a calcium-channel blocker (verapamil) on the morphology, cytoskeleton and collagenase activity of human skin fibroblasts. *Burns* **37,** 616–625 (2011).

18. Namani, R. Elastic Characterization of Transversely Isotropic Soft Materials by Dynamic Shear and Asymmetric Indentation. *J. Biomech. Eng.* **134,** 061004 (2012).

19. Chamis, C. C. Mechanics of Composite Materials: Past, Present, and Future. *J. Compos. Technol. Res.* **11,** 3–14 (1989).

20. Younes, R., Hallal, A., Fardoun, F. & Hajj, F. Comparative Review Study on Elastic Properties Modeling for Unidirectional Composite Materials. *Compos. Their Prop.* (2012). doi:10.5772/50362

21. Deguchi, S., Ohashi, T. & Sato, M. Tensile properties of single stress fibers isolated from cultured vascular smooth muscle cells. *J. Biomech.* **39,** 2603–2610 (2006).

22. Lu, L., Oswald, S. J., Ngu, H. & Yin, F. C.-P. Mechanical properties of actin stress fibers in living cells. *Biophys. J.* **95,** 6060–6071 (2008).

23. Hu, S. *et al.* Mechanical anisotropy of adherent cells probed by a three-dimensional magnetic twisting device. *AJP Cell Physiol.* **287,** C1184–C1191 (2004).

24. Buljak, V., Bocciarelli, M. & Maier, G. Mechanical characterization of anisotropic elasto-plastic materials by indentation curves only. *Meccanica* **49,** 1587–1599 (2014).
